# Genetic mapping of novel modifiers for *Apc*^*Min*^ induced intestinal polyps’ development using the genetic architecture power of the collaborative cross mice

**DOI:** 10.1186/s12864-021-07890-x

**Published:** 2021-07-22

**Authors:** Alexandra Dorman, Ilona Binenbaum, Hanifa J. Abu-Toamih Atamni, Aristotelis Chatziioannou, Ian Tomlinson, Richard Mott, Fuad A. Iraqi

**Affiliations:** 1 Department of Clinical Microbiology & Immunology, Sackler Faculty of Medicine, Ramat Aviv, 69978 Tel-Aviv, Israel; 2 Department of Biology, University of Patras, Patras, Greece; 3 Center of Systems Biology, Biomedical Research Foundation of the Academy of Athens, Athens, Greece; 4 Cancer Research UK Edinburgh Centre, Charles and Ethel Barr Chair of Cancer Research, University of Edinburgh, Edinburgh, UK; 5 Department of Genetics, University Collage of London, London, UK

**Keywords:** *Apc*^*Min*^, Colorectal cancer, Collaborative cross, Familial adenomatous polyposis, Genetic modifier, *Mom*s, Phenotyping, Recombinant inbred lines, QTL mapping, Candidate genes

## Abstract

**Background:**

Familial adenomatous polyposis is an inherited genetic disease, characterized by colorectal polyps. It is caused by inactivating mutations in the *Adenomatous polyposis coli* (*Apc*) gene. Mice carrying a nonsense mutation in the *Apc* gene at R850, which is designated *Apc*^*Min/+*^ (*Multiple intestinal neoplasia*), develop intestinal adenomas. Several genetic modifier loci of *Min* (*Mom*) were previously mapped, but so far, most of the underlying genes have not been identified. To identify novel modifier loci associated with *Apc*^*Min/+*^, we performed quantitative trait loci (QTL) analysis for polyp development using 49 F1 crosses between different Collaborative Cross (CC) lines and C57BL/6 J-*Apc*^*Min/+*^mice. The CC population is a genetic reference panel of recombinant inbred lines, each line independently descended from eight genetically diverse founder strains. C57BL/6 J-*Apc*^*Min/+*^ males were mated with females from 49 CC lines. F1 offspring were terminated at 23 weeks and polyp counts from three sub-regions (SB1–3) of small intestinal and colon were recorded.

**Results:**

The number of polyps in all these sub-regions and colon varied significantly between the different CC lines. At 95% genome-wide significance, we mapped nine novel QTL for variation in polyp number, with distinct QTL associated with each intestinal sub-region. QTL confidence intervals varied in width between 2.63–17.79 Mb. We extracted all genes in the mapped QTL at 90 and 95% CI levels using the BioInfoMiner online platform to extract, significantly enriched pathways and key linker genes, that act as regulatory and orchestrators of the phenotypic landscape associated with the *Apc*^*Min/+*^ mutation.

**Conclusions:**

Genomic structure of the CC lines has allowed us to identify novel modifiers and confirmed some of the previously mapped modifiers. Key genes involved mainly in metabolic and immunological processes were identified. Future steps in this analysis will be to identify regulatory elements – and possible epistatic effects – located in the mapped QTL.

**Supplementary Information:**

The online version contains supplementary material available at 10.1186/s12864-021-07890-x.

## Background

Colorectal cancer (CRC) is a complex genetic disease, with many genes influencing the expression of the disease [1]. Mutations in the *Adenomatous polyposis coli* (*Apc*) gene are relevant for > 80% of sporadic colorectal adenomas and inherited mutations in the *Apc* gene cause Familial Adenomatous Polyposis (FAP) syndrome [2]. However, the presence of a mutation in the *Apc* gene alone cannot explain the wide range of different clinical features of CRC, such as number/size/specific location and onset of polyp development. Environmental factors may contribute to these phenotypic differences, as do modify genes that modulate and regulate the expression and severity of the cancer development [3].

Experimental mouse models of cancer are ideal for examining the effects of genetic modifiers. Modifiers include loci that act, epistatically with known susceptibility loci, (i.e. a mutation in the *Apc* gene in CRC). Epistasis is difficult to detect in human genome wide association studies (GWAS), due to the very large sample size required. However, it is straightforward approach to engineer mutant mice in which a known susceptibility locus is altered to increase the risk of disease. By crossing the mutant into a population of mice with different genetic backgrounds of naturally occurring variation, it is theoretically possible to unearth the modifier loci.

Three decades ago, a mouse model for intestinal and colorectal cancer research was introduced by Moser et al. 1990; it was created by mutagenesis in germline of C57BL/6 (B6) mice strain and called *Min* (Multiple intestinal Neoplasia). Mice progeny from this mutated germline suffered from progressive anemia and had visible polyps in large and small intestine. This mice model has allowed further research of intestinal tumorgenesis [4].

Thus far, several genetic modifiers of *Min*, called Moms have been identified in various mice models, containing mutant versions of *Apc*^*Min/+*^ [5–8]. The genomic confidence intervals of most of the reported Moms, with few exceptions, were large, which limits the identification of candidate genes underlying these quantitative trait loci (QTL). So far, only two genes underlying two of these Moms were cloned, *Pla2g2a* for *Mom*1 and *Atp5a1* for *Mom*2, although their clinical significance is still not clear [7, 8].

In this study, we used a mouse panel formed by crossing *Apc*^*Min/+*^mice with Collaborative Cross (CC) mice [9, 10], in order to map novel Moms. Nowadays, the completed CC comprises a set of ~ 70 Recombinant Inbred Lines (RILs) that were created by full reciprocal matings between 8 different mice strains (the CC founders). These 8 founder strains are genetically diverse, including 5 common laboratory strains: A/J, C57BL/6 J, 129S1/SvImJ, NOD/LtJ, NZO/HiLtJ, and 3 wild-derived strains: CAST/Ei, PWK/PhJ, and WSB/EiJ [11].

The advantages of using CC F1 hybrids for modifier mapping include the numerous genetic variants segregating in the population (there are over 50 million SNPs) [12, 13] e.g. only ~ 4.4 million SNPs segregate between the founders of the BXD panel of RILs [14], and the relatively high level of recombination events compared to two-parent mouse RILs. The three wild-derived founders of the CC represent different subspecies, *M.m castaneus, M.m musculus* and *M.m. domesticus*, and contribute many novel sequence variants, not segregating among classical laboratory strains descended from *M.m. domesticus* [13–15]. Many QTLs mapped in CC mice involve allelic contrasts between the wild-derived and laboratory strains [16, 17]. Previous simulation of QTL mapping in CC mice has shown that confidence intervals are typically shorter than 1 Mb [18], and our recent results from variety of studies have shown that it was possible to map the QTL even within less than 1 MB genomic intervals [16, 17].

## Methods

### Generation of CC- B/6-min mice

In total, 957 F1 mice were produced by a cross of females from 49 CC lines to C57B/6 J-*Apc*^*Min*/+^ males and after PCR analysis for Min genotype, 402 F1 CC-C57BL/6-*Apc*^*Min*/+^ (CC-B/6-*Apc*^*Min*/+^) mice were identified and included in the study for further assessment and analysis. Table 1 shows the list of all the used 49 CC lines and number of mice used from each line. The CC mouse lines were developed and maintained at conventional environmental conditions at the small animal facility of Tel-Aviv University (TAU) and were between generations of G10 to G28 of inbreeding by full-sib mating as, fully described, earlier [11]. The C57BL/6 J- *Apc*^*Min*/+^ mouse line was purchased from the Jackson Laboratory (Bar Harbor, Maine, USA). All experimental mice and protocols were approved by the Institutional Animal Care and Use Committee (IACUC) of Tel-Aviv University (TAU), approval numbers: M-08-075; M-12-024, which adheres to the Israeli guidelines that follow NIH/USA animal care and use protocols.

**Table 1:** Summary of number of all the used male and female mice of the 49 different lines of the Collaborativ Cross mouse population. # shows the 49 lines; TAU CC lines, shows the TAU designation i.e. Ilxxxx; JAX CCxxx shows the current international CC designation available at JAX laboratory; Male, shows the number of used male mice per line; Female, shows the number of used females per line

#	TAU CC lines	JAX CCxxx	Male	Female
1	IL72	CC037	4	7
2	IL111		5	5
3	IL188	CC004	11	5
4	IL211	CC005	1	3
5	IL219		4	3
6	IL519		5	2
7	IL521	CC072	9	5
8	IL534		3	4
9	IL557	CC040	4	4
10	IL611		8	6
11	IL670		3	0
12	IL688		7	3
13	IL711		3	3
14	IL785		7	2
15	IL1052		2	3
16	IL1061		5	5
17	IL1156		7	4
18	IL1286		0	1
19	IL1300		7	8
20	IL1379		2	2
21	IL1488		5	6
22	IL1513		2	8
23	IL1912	CC051	5	5
24	IL2011		4	3
25	IL2126	CC078	8	8
26	IL2146		3	2
27	IL2156		3	6
28	IL2288		0	1
29	IL2391		2	1
30	IL2438		5	4
31	IL2439		3	3
32	IL2462		8	3
33	IL2478		0	3
34	IL2513	CC019	4	2
35	IL2573		7	8
36	IL2680		1	4
37	IL2689		6	3
38	IL2693		1	0
39	IL2750	CC006	8	3
40	IL3348		4	2
41	IL3438	CC084	3	7
42	IL3480		1	1
43	IL3575		2	3
44	IL3912	CC059	7	4
45	IL4052		7	7
46	IL4141	CC041	6	2
47	IL4156		2	4
48	IL4438		0	2
49	IL4457		11	7
**Total mice**			**215**	**187**

All experimental mice were weaned at age of 3 weeks old, housed separately by sex, maximum five mice per cage, with standard rodents’ chow diet (TD.2018SC, Teklad Global, Harlan Inc., Madison, WI, USA, containing % Kcal from Fat 18%, Protein 24%, and Carbohydrates 58%) and water ad libitum. All animals housed in TAU animal facility at conventional open environment conditions, in clean polycarbonate cages with stainless metal covers, and bedded with wood shavings. A Light: dark cycles of 12:12 h, and constant room temperature of 22^0^_c_ (±2). Due to genetic variations between the CC lines, breeding rate, number and sex of litters in each cycle might vary.

### Genotyping of CC-B/6-min mice

At 4 weeks old, 0.5 cm tail biopsies were collected from CC-X B/6- *Apc*^*Min*/+^ mice and DNA extracted by NaOH boiling protocol [19]. Mice were genotyped by Polymerase chain reaction (PCR) for the *Apc*^*Min*/+^ mutant allele, using the primers: MAPC-min (TTCTGAGAAAGACAGAAGTTA), MAPC-15 (TTCCACTTTGGCATAAGG), and MAPC-9 (GCCATCCCTTCACGT). For *Apc* wild type alleles, we used the primers MAPC-15 and MAPC-9, while for the mutant allele we used MAPC-min and MAPC-15 primers [20]. For later identification each mouse was labeled with ear clipping.

### Intestinal preparations for polyps count

At the terminal point of the experiment (when mice were 23 weeks old), 402 mice (215 males and 187 females), from 49 CC-B/6-*Apc*^*Min*/+^ lines (*n* = 1–18 mice per line) were sacrificed by CO2 protocol. Subsequently, small intestines and colons were extracted and washed with Phosphate Buffered Saline (PBS). The small intestines were divided into three segments (SB1-proximal, SB2-middle, and SB3-distal), and the colon was kept as a whole and spread over 3 mm paper. The intestines were fixed in 10% Neutral Buffered Formalin (NBF) overnight and stained by 0.02% methylene blue. The samples were then examined by binocular. The counts and sizes (< 1 mm, 1-2 mm, 2-3 mm, > 3 mm) of polyps in each of the four intestinal sub-regions were recorded as described in Rudling et al. 2006 [21].

### Data analysis

Initial statistical analyses were performed using a statistical software package SPSS version 19. One-way Analysis of variance (ANOVA) was performed to test the significance levels of variations in total polyp counts between the different CC-B/6-Min crosses.

### CC lines genotype data

High molecular genomic DNA of the CC lines were initially genotyped with the mouse diversity array (MDA), which consists of 620,000 SNPs [22] and re-genotyped by mouse universal genotype array (MUGA-7500 markers) and eventually with MegaMuga (77,800 markers) SNP arrays to confirm their genotype status [12]. The genotype database used in this study is, publically available at: http://mtweb.cs.ucl.ac.uk/mus/www/preCC/CC-2018/LIFTOVER/CONDENSED/.

Data analysis was performed using the statistical software R (R Development Core Team 2009), including the R package HAPPY.HBREM [23].

### Reconstruction of CC ancestral genome mosaics

We removed SNPs with heterozygous or missing genotypes in the 8 CC founders, or were not in common between the arrays, leaving 170,935 SNPs. The SNPs were mapped onto build 37 of the mouse genome. We reconstructed the genome mosaic of each CC line in terms of the eight CC founders using a hidden Markov Model HAPPY ([23] across the genotypes to compute probabilities of descent from founders, setting the generation parameter to g = 7. To allow for genotyping error, we configured the HMM to allow a small probability of 0.001 that any founder was consistent with any SNP allele. The HAPPY HMM computed a descent probability distribution for each of the 170 k SNP intervals, which we reduced to 8533 intervals by averaging the matrices in groups of *n* = 20 consecutive SNPs. This reduction reduced further the effects of genotyping error and made analyses faster. Mean heterozygosity was computed across each window of 20 SNPs.

The locus-specific fraction of CC lines carrying each of the founders was estimated by summing the HMM posterior probabilities at each interval across all lines. Genome wide thresholds for significance were computed by permuting the identities of the founders separately within each line, then recomputing the locus-specific fractions and recording the genome wide maximum and minimum fractions in the permuted data. This process was repeated 200 times to estimate the upper and lower thresholds exceeded in 10% of permutations.

### QTL analysis

The genome of each CC line is a mosaic of the inbred founders, which we reconstructed using a hidden Markov model implemented in the HAPPY R package across the genotypes to compute probabilities of descent from the founders [13, 23]. The presence of a QTL at a given locus was tested using the probabilities of descent from each founder calculated through HAPPY and testing for association between the founder haplotype at each locus and the median polyp count within each CC line, using multiple linear regression. Sex was included as a covariate. QTL effect sizes were estimated as the proportion of the log-likelihood explained by the locus effects at the QTL. Genome-wide significance was estimated by permutation, where the CC line labels were permuted between the phenotypes. Permutation-based false discovery rate (FDR) was calculated for a given *P*-value threshold, following the formula: (expected number of false discoveries)/ (number of observed discoveries).

### Testing sequence variation segregating between the CC founders

Except for a small number of *de-novo* mutations arising during breeding, all sequence variants segregating in the CC should also segregate in the CC founders. Therefore we use the merge analysis methodology [24] to test which variants under a QTL peak were compatible with the pattern of action at the QTL. A variant with *A* alleles inside the locus *L* merges the 8 CC founders into *A < 8* groups according to whether they share the same allele at the variant (*A* = 2 in the case of SNPs). This merging is characterized by an 8x*A* merge matrix *M*_*sa*_ defined to be 1 when strain *s* carries allele *a,* and 0 otherwise. The effect of this merging is tested by comparing the fit of the QTL model above with one in which the *N*x8 matrix *X*_*Lis*_ is replaced by the *N*x*A* matrix *Z*_*ia*_ = Σ_*s*_ *X*_*Lis*_ *M*_*sa*_. We use the Perlegen SNP database to test sequence variants globally and the Sanger SNP database for individual genes. This approach was, successfully applied in our previous studies [16, 17, 24].

### Estimation of QTL confidence intervals

The confidence intervals of the QTL were estimated through simulation of a QTL with a similar logP and strain effects in the neighborhood (5 Mb) of the observed QTL peak, using a similar approach as presented in our previous studies [16, 17] to take into account local patterns of linkage disequilibrium. Briefly, accurate estimates of QTL mapping resolution should take into account local patterns of linkage disequilibrium. We devised a method that preserved the genotypes of the data, whilst simulating survival times caused by a QTL in the neighborhood (5 Mb) of the observed QTL peak, and with a similar logP to that observed. We first extracted the parameter estimates ${\hat{\beta}}_s$ and residuals ${\hat{r}}_i$ of the fitted polyp counts model at the QTL peak. Let ${\hat{t}}_i$ be a random permutation of ${\hat{r}}_i$. Then in a marker interval *K* within 5 Mb of the QTL peak *L* we simulated a set of survival times *Z*_*iK*_ caused by a QTL at *K* by substituting the parameter estimates and permuted residuals:

$$ {Z}_{iK}=t\hat{\mkern6mu} i\kern0.5em \exp \left(\upmu \hat{\mkern6mu} +\Sigma s\ {X}_{Kis}\upbeta \hat{\mkern6mu} s\right) $$

We then rescanned the region and found the interval with the highest logP. We simulated 1000 QTLs at each interval K and estimated the *p*% CI from interval containing p% of the simulated local maxima.

### Founder effects

Except for a small number of de-novo mutations arising during breeding, all sequence variants segregating in the CC lines should also segregate in the CC founders. The founder strain trait effects at each QTL were shown relatively to WSB/EiJ, using a similar approach as presented in our previous studies [16, 17]. Briefly, except for a small number of *de-novo* mutations arising during breeding, all sequence variants segregating in the CC should also segregate in the CC founders. Therefore, we use the merge analysis methodology [24] to test which variants under a QTL peak were compatible with the pattern of action at the QTL. A variant with *A* alleles inside the locus *L* merges the 8 CC founders into *A < 8* groups according to whether they share the same allele at the variant (*A* = 2 in the case of SNPs). This merging is characterized by an 8x*A* merge matrix *M*_*sa*_ defined to be 1 when strain *s* carries allele *a,* and 0 otherwise. The effect of this merging is tested by comparing the fit of the QTL model above with one in which the *N*x8 matrix *X*_*Lis*_ is replaced by the *N*x*A* matrix *Z*_*ia*_ = Σ_*s*_ *X*_*Lis*_ *M*_*sa*_. We use the Perlegen SNP database (http://mouse.perlegen.com/mouse/download.html) to test sequence variants globally and the Sanger mouse genomes database (http://www.sanger.ac.uk/resources/mouse/genomes/) for individual genes.

Within the QTLs we classified the sequence variants according to the genome annotation as repetitive, intergenic, upstream, downstream, UTR, intronic or coding. We then classified variants according to whether their merge logP was greater or less than the corresponding haplotype-based logP. The enrichment of variants with high logP values within each category was computed.

### List of suggested candidate genes

We used the SNP tools package in R, and the MGI database (http://www.informatics.jax.org) to find all the genes in the 95% confidence interval for each QTL. We focused on protein-coding genes in these regions, but also non-coding RNA genes, such as miRNA loci. Also, if the 3′ UTR or the 5′ UTR of a gene were inside the interval then we included the gene in our list. We used these candidate gene lists as an input for BioInfoMiner.

### Functional analysis with BioInfoMiner

We performed functional pathway analysis using BioInfoMiner [25]. BioInfoMiner (https://bioinfominer.com) performs statistical and network analysis on biological hierarchical vocabularies to detect and rank significantly enriched processes and the underlying hub genes involved in these processes. For our analysis, we used Gene Ontology (GO) [26], Reactome [27] and MGI Mammalian Phenotype (MGI) [28]. The BioInfoMiner algorithm maps the genes in the supplied gene list to a semantic network created from ontological data, corrected through AI-inspired semantic network pruning and clustering and then prioritizes the genes based on the topological properties of the thus corrected network. This analysis prioritized genes with central functional and regulatory roles in enriched processes, underlying the studied phenotype. The correction for potential semantic inconsistencies on the selected ontological scheme and bias mitigation regarding the different depth of the branches of the semantic tree, as a result of differences in knowledge representation for distinct scientific concepts, was performed by restoring the order of the resolution of annotation of each gene with its ancestral ontological terms.

## Results

### Polyp counts

**Fig. 1:**
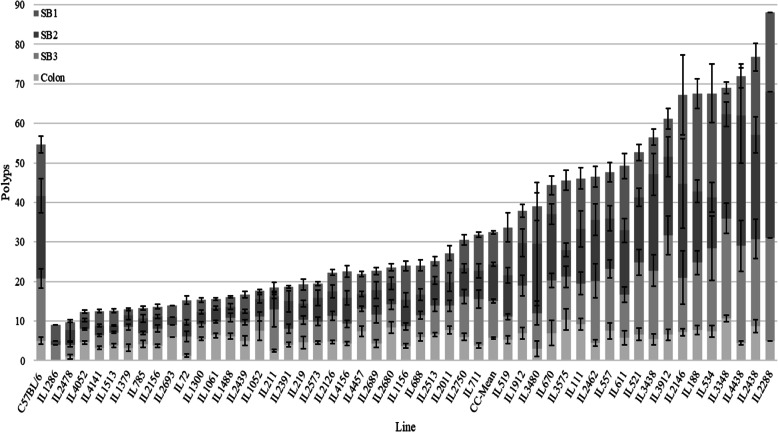
Polyp’s count (±SE) inSB1, SB2, SB3, and Colon of CC-F1 mice crosses at the age of 23 wks. Old (terminal time point). Data analysis of 49 CC-B/6-Min F1 hybrid lines (*n* = 1–18 mice/line) and C57BL/6-Minstrain (4 mice). The Y-axis represents the number of polyps; X-axis represents different APC-min F1 hybrid lines. First column represents C57BL/6 carrying the ApcMin/+ mutation (first column) and mean of the CC-B/6-Min population. Data analyzed by One-way Analysis of Variation (ANOVA), *p-value< 0.05

We mapped QTL modifiers of *Apc*^*Min*/+^ based on polyp counts in the small intestine and colon, in 49 CC-B/6-*Apc*^*Min*/+^ lines at 23 weeks old (*n* = 402 mice) (see Fig. 1). The overall population mean of total polyp counts was 32.48 ± 1.36 polyps, ranging widely from 9 polyps (IL1286) to 88 polyps (IL2288). Based on one-way ANOVA, significant variation (*p* < 0.01) was found between different 49 CC-B/6-*Apc*^*Min*/+^ lines in their total counted polyps. Polyp counts were approximately normally distributed, suggesting the intervention of numerous genetic and environmental factors in this trait. The mean of polyp number for the parental line B/6-*Apc*^*Min*/+^(*n* = 5) (first column Fig. 1) was 64.25 ± 6.65 polyps. The majority of CC-B/6-*Apc*^*Min*/+^ lines (30/49, 61%) had lower polyp counts compared to the B/6-*Apc*^*Min*/+^ parental line suggesting that resistant alleles for intestinal tumorigenesis segregate among the CC lines. We also investigated if different segments of the intestine exhibited differential polyp distribution and different genetic architectures. The small intestine was subdivided into 3 sections (small intestine proximal-SB1, middle-SB2, and distal-SB3), and the colon was treated separately. Overall polyps were distributed approximately equally between 3 segments of the small intestine: SB1 with 8.12 ± 0.45 polyps (25%), SB2 with 9.25 ± 0.53 (28.48%), SB3 with 9.37 ± 0.48 (28.85%) and the colon was with 5.7 ± 0.19 (17.47%).

### QTL analysis

QTL analysis using HAPPY [16, 17, 23] was performed for polyp count traits sub-divided into three parts of the small intestinal tract (SB1, SB2, and SB3) and colon, for the 402 mice in 49 CC-B/6- *Apc*^*Min/+*^ F1 crosses, including males and females. Nine significant QTLs at the genome-wide significance threshold of 90% (i.e. where < 10% of permutations had a genome-wide maximum exceeding an observed QTL score) were detected (Table 2). Five of these QTLs were significant at the more stringent 95% level of genome-wide significance.

**Table 2:** Genomic location of the significant Quantitative Trait Loci (QTL) at 90 and 95% genome wide significant thresholds associated with polyp counts in SB1, SB2, SB3, Colon and total polyps in the entire intestines (SB1–3 and colon) regions of different CC lines. QTL associated with polyp counts detected on different chromosomal regions. Experiment-wide thresholds of significance at *P% of 50, 90 and 95% levels are presented for each trait, accordingly

Trait	logP		Chrs	QTL	Peak (Mb)	CI 50% Size (Mb) [Genes]	CI 90% Size (Mb) [Genes]	CI 95% Size (Mb) [Genes]
	*90%	**95%						
SB1	3.71	4.43	**Chr3**	*Mom*19**	13.839	13.434–14.321 (0.88) [12]	11.203–17.131 (5.93) [49]	9.902–19.627 (9.72) [104]
			**Chr12**	*Mom*20*	111.371	110.004–113.284 (3.28) [104]	103.706–117.303 (13.60) [610]	102.018–118.857 (16.84) [670]
SB2	3.83	4.11	**Chr10**	*Mom*21**	18.805	17.208–20.747 (3.54) [46]	9.550–27.338 (17.79) [234]	8.902–28.471 (19.57) [245]
			**Chr16**	*Mom*22**	53.511	52.785–56.096 (3.31) [34]	48.038–62.078 (14.04) [186]	45.522–63.132 (17.61) [226]
				*Mom*23**	73.216	72.224–73.812 (1.59) [9]	69.722–76.406 (6.68) [51]	68.716–78.148 (9.43) [73]
SB3	3.90	4.20	**Chr6**	*Mom*24**	146.203	145.502–146.376 (0.87) [8]	140.899–147.303 (6.40) [103]	138.051–147.806 (9.76) [135]
			**Chr12**	*Mom*25**	113.449	112.966–113.893 (0.93) [96]	110.997–115.709 (4.71) [299]	109.825–116.663 (6.84) [375]
			**Chr9**	*Mom*26*	37.552	35.326–39.645 (4.32) [176]	32.692–42.502 (9.81) [271]	32.557–42.557 (10.00) [273]
			**Chr10**	*Mom*21*	18.805	16.268–20.395 (4.13) [48]	9.921–25.465 (15.54) [211]	8.950–27.582 (18.63) [238]
Colon	3.87	4.19	**Chr6**	*Mom*27**	35.915	35.651–36.331 (0.68) [3]	35.031–37.665 (2.63) [27]	34.720–38.392 (3.67) [59]
Total polyps	3.86	4.23	**Chr12**	*Mom*20**	111.636	111.349–112.016 (0.67) [26]	109.935–113.616 (3.68) [156]	109.525–113.920 (4.39) [284]
			**Chr16**	*Mom*22**	53.489	51.882–56.475 (4.59) [42]	45.709–62.530 (16.82) [211]	44.055–63.294 (19.24) [258]
				*Mom*23**	73.556	72.068–74.972 (2.90) [18]	68.468–80.424 (11.96) [93]	65.848–83.013 (17.16) [119]

**95%, *90% levels of genome wide significance thresholds

**Fig. 2:**
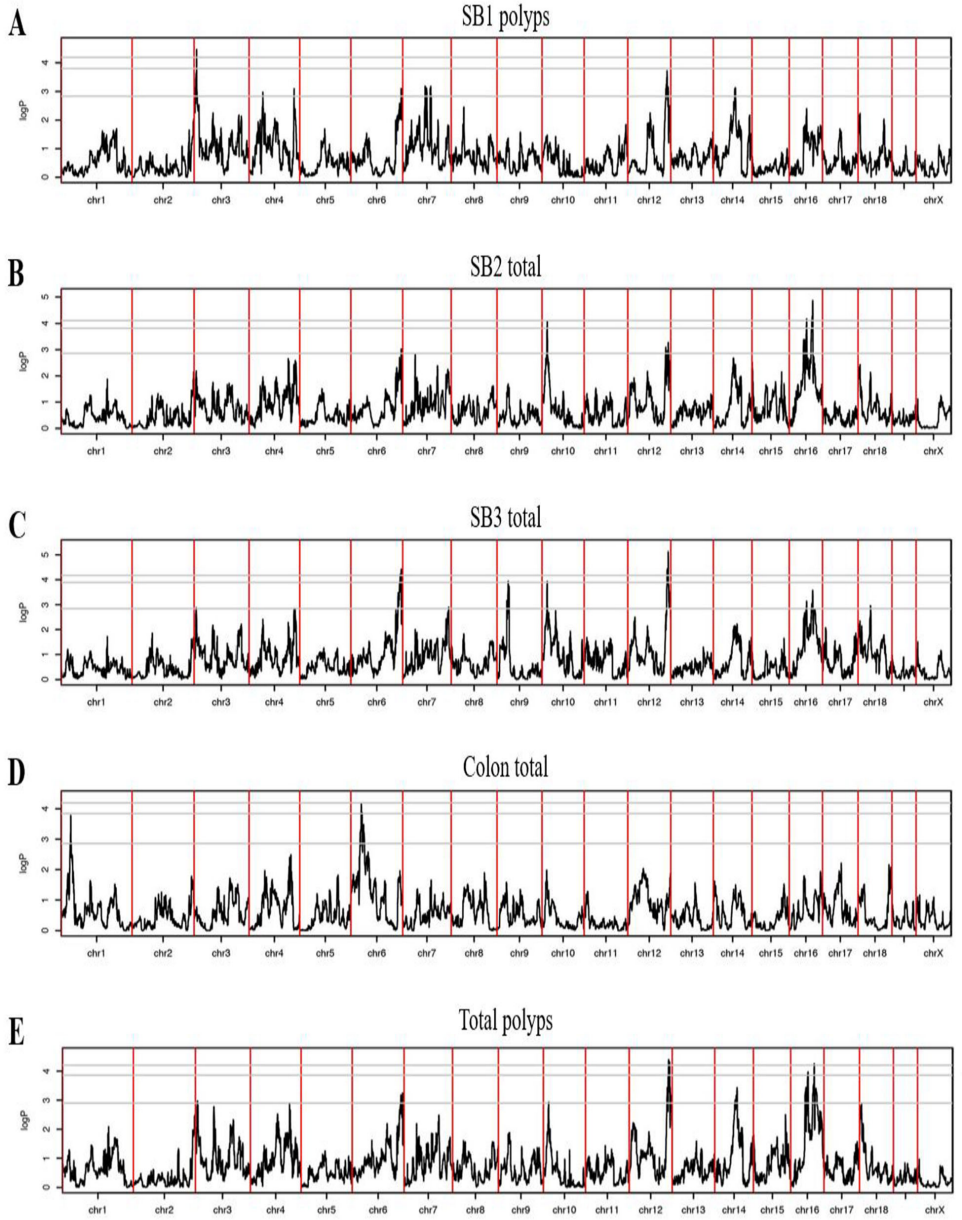
Genome scans for counted polyps with significant QTLs. **A** Polyps counted in SB1. **B** Polyps counted in SB2. **C** Polyps counted in SB3. **D** Polyps counted in the colon. **E** Total counted polyps. The X-axis represents the 19 mouse chromosomes and the position of mapped QTLs. Y-axis represents the logP of the test of association between locus and polyps count

In the proximal section of the small intestine, SB1, (Fig. 2A), a significant QTL (95%) was found on chromosome 3, peak at 13.839 Mb, logP =4.43, designated *Mom19*. Another significant QTL (90%) was found on chromosome 12, peak at 111.37 Mb, logP = 3.71, designated *Mom*20. For SB2, (Fig. 2B), a significant QTL (95%) was found on chromosome 10, peak at 18.805 Mb, logP =4.11, designated *Mom*21. Additionally, two well-separated significant QTLs (95%) for SB2 were found on chromosome 16, peak at 53.51 Mb (*Mom*22) and 73.216 Mb (*Mom*23), logP > 4. For SB3, (Fig. 2C), two significant QTLs (95%) were found on chromosome 6 and chromosome 12, peak at 146.203 Mb (*Mom*24) and 113.449 Mb (*Mom*25) respectively, logP > 4.2. Further, two QTLs (90%) were found on chromosome 9, peak at 37.55 Mb, logP = 3.9, on chromosome 10 same location as *Mom*21. For polyp’s count in colon, Fig. 2D, a solo significant QTL (95%) was found on chromosome 6, peak at 35.91 Mb, logP = 4.19, designated *Mom*27. For total polyp counts, Fig. 2E, a significant QTL (95%) was mapped to same locations of *Mom*20, *Mom*22 and *Mom*23.

In summary, nine distinct and novel QTLs at 90 and 95% genome-wide significant thresholds levels (GWSL). These QTLs were designated as modifiers of Min gene (*Mom*) numbers 19–27, respectively, presented in Table 2 the location of the peak, the interval and number of genes within each of QTLs.

Finally, we lowered the genome-wide significant thresholds to the 50% level (i.e. where there was a probability of 50% that a QTL exceeding the threshold was a false positive), to identify potential candidate QTLs, which might be genome-wide significant in a larger and more powerful mapping populations. Table 3 summarizes all the mapped QTL at 50% threshold level, their chromosomal locations, 90 and 95% confidence intervals and number of genes identified within these intervals. The analysis has detected four QTL associated with SB1 trait, where two of them were mapped at different positions on chromosome 7 (peaks at 87.273 and 103,732 Mb), one QTL mapped at chromosome 6 (peak at 145.956 Mb), and one on chromosome 14 (peak at 73.794 Mb).

**Table 3:** Chromosomal locations of the mapped Quantitative Trait Loci (QTL) at 50% genome wide significant threshold level and found to be associated with polyp counts in SB1, SB2, SB3, Colon and total polyps in the entire intestines (SB1–3 and colon) regions of different CC lines. QTL associated with polyp counts detected on different chromosomal regions. Experiment- confidence intervals (CI) of significance at *P% of 90 and 95% levels are presented for each trait, accordingly

Trait	Chromosome location	Peak	CI 90% Size (Mb) [Genes]	CI 95% Size (Mb) [Genes]
SB1	chr7	87.273	79.392–92.031	77.912–93.918
			(12.639)	(16.006)
			[260]	[309]
	chr7	103.731	94.877–110.206	93.967–112.342
			(15.329)	(18.375)
			[582]	[615]
	chr6	145.956	137.764–147.736	136.531–148.381
			(9.972)	(11.85)
			[138]	[167]
	chr14	73.794	64.12–82.311	63.807–83.451
			(18.191)	(19.644)
			[296]	[312]
SB2	chr6	146.284	138.715–147.765	137.334–148.218
			(9.05)	(10.884)
			[129]	[147]
	chr12	113.669	104.039119.56	103.67–120.723
			(15.521)	(17.053)
			[620]	[642]
SB3	chr3	12.197	6.161–21.878	4.711–22.198
			(15.717)	(17.487)
			[174]	[190]
	chr4	140.994	131.573–150.502	131.067–150.94
			(18.929)	(19.873)
			[667]	[677]
	chr7	145.941	138.321–152.901	136.883–154.414
			(14.58)	(17.531)
			[244]	[254]
	chr16	73.271	68.142–79.809	65.692–82.693
			(11.667)	(17.001)
			[271]	[370]
	chr16	53.511	43.867–63.054	43.524–63.477
			(19.187)	(19.953)
			[266]	[272]
	chr18	40.491	30.571–49.528	30.483–50.356
			(18.957)	(19.873)
			[379]	[392]
Colon	chr1	34.282	33.429–35.455	32.859–35.727
			(2.026)	(2.868)
			[35]	[43]
Total polyps	chr3	11.823	3.155–21.794	2.322–21.835
			(18.639)	(19.513)
			[199]	[199]
	chr4	126.165	124.866–127.097	123.887–127.464
			(2.231)	(3.577)
			[74]	[119]
	chr6	146.233	138.363–147.629	137.058–148.081
			(9.266)	(11.023)
			[130]	[146]
	chr10	19.133	10.259–27.523	9.325–28.692
			(17.264)	(19.367)
			[228]	[247]
	chr14	74.654	67.9751–78.268	66.108–82.316
			(10.2929)	(16.208)
			[176]	[258]
	chr18	10.631	6.347–16.314	5.249–17.996
			(9.967)	(12.747)
			[149]	[174]

Two QTL were detected with SB2 trait and mapped on chromosomes 6 and 12 at positions of 146.284 and 113.669 Mb, respectively. Six QTL were detected with SB3 trait, while two of them were mapped at different positions on chromosome 16 (peaks at 53.511 and 73.271 Mb), a single QTL was mapped on chromosomes 3, 4, 7 and 18 at positions 12.197, 140.994, 145.941 and 40.491, respectively. One QTL was detected with polyps in the colon and mapped at chromosome 1 its peak was located at 34.282 Mb. Finally, six QTL were detected with total polyp’s trait, and mapped at chromosomes 3, 4, 6, 10, 14 and 18, and its peaks were located at 11.832, 126.165, 146.233, 19.133, 76.654 and 10.631, respectively. These 90 and 95% confidence intervals of the identified QTL were ranged between 10 and 20 Mb, and number of genes identified within these intervals were ranged between 150 to 670.

### Founder effects

The effects of each founder haplotype on polyp counts for the mapped QTLs were evaluated as deviation relative to the WSB/EiJ parental strain, which was arbitrary assigned the baseline zero effect. All the data presented in Table 4. For *Mom*19 there were slight positive effects on poly counts for CAST/EiJ, NZO/HILtJ, 129S1/SvImJ strains and minimal negative effects for A/J, C57BL/6 J, NOD/LtJ, and PWK/PhJ. For *Mom*20 all founder strains have positive effects except A/J and C57BL/6 J. For *Mom*21 all the founder strains contributed a positive effect on polyps count (i.e. this QTL involved a contrast between WSB/eiJ vs the rest). For *Mom*22and *Mom*23 all the founder strains except PWK/PhJ contributed positive effects. For *Mom*24 positive effects were seen in all the founder strains, except CAST/EiJ and 129S1/SvImJ. For *Mom*25 only A/J and C57BL/6 J strain had a minor negative effect on polyps count. For *Mom*26 and *Mom*27 C57BL/6 J, CAST/EiJ and 129S1/SvImJ had a negative effect.

**Table 4:** The estimated strain effects on polyp count for the 8 CC founder strains for each of the mapped Quantitative Trait Loci (QTL) *Mom*19-*Mom*27, which were mapped at 90 and 95% genome wide significant thresholds levels. Effects are shown as deviations relative to WSB/EiJ, which is arbitrarily assigned the trait effect

	***A/J***	***C57BL/6 J***	***CAST/EiJ***	***NOD/LtJ***	***NZO/HILtJ***	***PWK/PhJ***	***129S1/SvImJ***
***Mom*19**	−2.50	−1.47	0.10	−0.68	3.11	−1.66	3.52
***Mom*20**	−5.07	−1.64	6.63	17.47	11.36	10.59	7.62
***Mom*21**	3.38	2.01	4.54	0.68	0.56	0.72	6.10
***Mom*22**	14.90	8.07	10.75	13.64	32.78	−1.29	1.62
***Mom*23**	30.35	10.43	7.76	8.71	27.84	−0.46	10.10
***Mom*24**	12.69	4.62	−1.72	1.86	3.52	0.88	−1.93
***Mom*25**	−3.66	−0.09	1.98	6.58	4.66	2.25	1.95
***Mom*26**	3.36	−1.09	−2.78	4.89	0.16	0.31	−2.87
***Mom*27**	0.28	−0.26	−0.89	0.29	1.15	6.03	−0.29

### Merge analysis

**Fig. 3:**
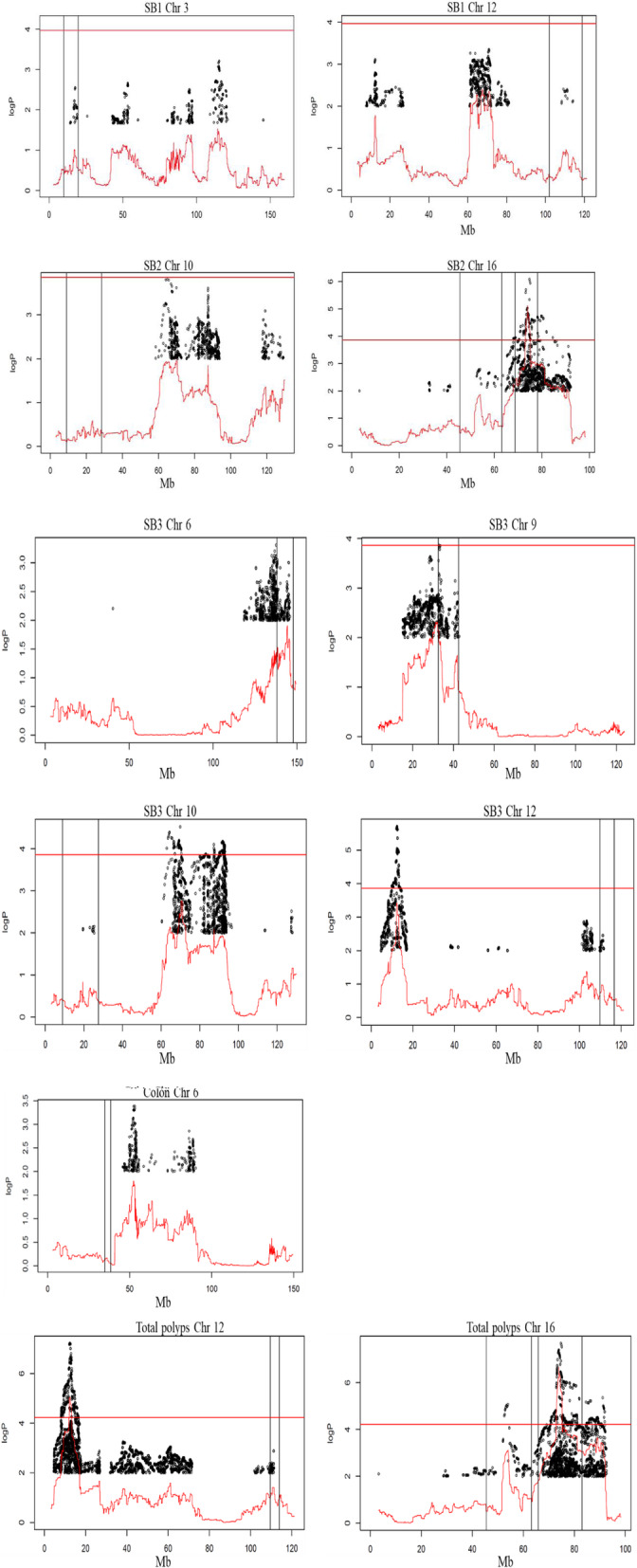
Merge analysis of sequence variants around the mapped QTL, for susceptibility to polyp development in the CC lines. The X-axis represents genomic location, the Y-axis represents the logP of the test of association between locus and polyps count. The continuous red line is the genome scan of different mapped QTL in Fig. 2. The black dots are the results of merge analysis tests of sequence variants segregating in the 8 founders of the CC lines

The haplotype QTL analysis was then refined by merge analysis in order to identify SNPs within each QTL whose strain distribution patterns among the founder strains were consistent with the patterns of action at the QTL. Results are presented in Fig. 3. In every plot two vertical lines that delineate the location of the mapped QTL. In three plots of Fig. 3 of SB2 Chr16, SB3 Chr6 and SB3 Chr9, the locations of SNPs were mapped at the same interval as the mapped QTLs, *Mom*23, *Mom*24, *Mom*26. For SB1, we did not find any SNPs with logP> 4 within the mapped QTLs, suggesting that the effect was not driven by a single biallelic variant but instead was haplotype-based. For SB2 significant SNPs were found on chromosome 16, same location as *Mom*23. For SB3 significant SNPs were found on chromosome 9 same location as *Mom*26, on chromosomes 10 and 12 significant SNPs were found but outside of mapped QTLs. It is interesting to note that significant SNPs on chromosome 10 were mapped to the same location as in the previously mapped *Mom*17 [29]. For colon polyps count, unfortunately, we did not find significant SNPs within the mapped QTLs. For total polys count, significant SNPs were found on chromosome 12 but outside of mapped QTL interval, on chromosome 16 significant SNPs were found on same locations as *Moms*22 and 23.

### Candidate genes

Our results have shown that based on the mapped QTL analysis, we have 1862 unique genes in the intervals of all the mapped QTL. When the Bioinfominer analysis was performed, while searching for genes based on functional pathway analysis, which prioritized genes with central functional and regulatory roles in enriched processes, underlying the studied phenotype, we identified 411 unique genes related to the studied traits (supplement 1).

Merge analysis identified candidate functional SNPs that may play a role in each QTL, some of which were outside the haplotype-based QTL regions. We used the MGI database to select genes nearest these SNPs. Being outside of the original QTL intervals, some of the are not in the original candidate gene lists based on the BioInfoMiner analysis. We found a total of 123 candidate genes, presented in Table 5, but only two of them are topic relevant. These two genes are mapped to a SNP on chromosome 10 which is associated with SB3 polyp counts. The first is colon tumor susceptibility 9 (*Scc9*) locus, which was previously mapped in BALB/c × CcS crosses [30], and the second gene is angiogenesis *by VEGF QTL 1(Angvq1),* which was mapped by using BXD strains [31].

**Table 5:** Candidate gene list identified within the mapped QTL, *Mom*19–27, based on merge analysis and using MGI data base

Trait	Chromosome	Mom	Gene	
SB2	16	*Mom 22 / Mom 23*	*Eih3*	ethanol induced hypothermia 3
		*Mom 22 / Mom 23*	*Bwtn1*	body weight at necropsy 1
		*Mom 22 / Mom 23*	*Aod1a*	autoimmune ovarian dysgenesis 1a
		*Mom 22 / Mom 23*	*Skts-fp3*	skin tumor susceptibility in FVB and PWK 3
		*Mom 22 / Mom 23*	*Eae41*	experimental allergic encephalomyelitis susceptibility 41
		*Mom 22 / Mom 23*	*Diobq*	diet-induced obesity QTL
		*Mom 22 / Mom 23*	*Dice1b*	determination of interleukin 4 commitment 1b
		*Mom 23*	*Eae11*	experimental allergic encephalomyelitis susceptibility 11
		*Mom 23*	*Lith14*	lithogenic gene 14
		*Mom 23*	*Pod*	plasticity of ocular dominance
			*Sluc27*	susceptibility to lung cancer 27
			*Etia*	ethanol induced activation
			*Bpq9*	blood pressure QTL 9
			*Tgq28*	triglyceride QTL 28
			*Pgia10*	proteoglycan induced arthritis 10
SB3	9	*Mom 26*	*Sles4*	systemic lupus erythmatosus suppressor 4
		*Mom 26*	*Bmiq8*	body mass index QTL 8
		*Mom 26*	*Obq5*	obesity QTL 5
		*Mom 26*	*Igan3*	IgA nephropathy 3
		*Mom 26*	*Elnv*	epilepsy naive
		*Mom 26*	*V125Dq7*	vitamin D active form serum level QTL 7
		*Mom 26*	*V125Dq8*	vitamin D active form serum level QTL 8
SB3	10	*Mom 21*	*Obsty3*	obesity 3
		*Mom 21*	*Sysbp1*	systolic blood pressure 1
		*Mom 21*	*W3q11*	weight 3 weeks QTL 11
			*W6q6*	weight 6 weeks QTL 6
		*Mom 21*	*W10q5*	weight 10 weeks QTL 5
		*Mom 21*	*Egq7*	early growth QTL 7
		*Mom 21*	*Hrtq3*	heart weight QTL 3
		*Mom 21*	*Kidpq2*	kidney weight percentage QTL 2
		*Mom 21*	*Lvrq4*	liver weight QTL 4
		*Mom 21*	*Scfpq1*	subcutaneous fat pad percentage QTL 1
			*Scc9*	colon tumor susceptibility 9
			*Eae34*	experimental allergic encephalomyelitis susceptibility 34
			*Lmblgq4*	limb length QTL 4
			*Lmblgq4*	limb length QTL 4
			*Angvq1*	angiogenesis by VEGF QTL 1
			*Phl2*	progressive hearing loss 2
			*Cia8*	collagen induced arthritis QTL 8
			*Wght9*	weight 9
			*Bbaa18*	B.burgdorferi-associated arthritis 18
			*Skull14*	skull morphology 14
			*Lmb4*	lupus in MRL and B6 F2 cross, QTL 4
			*Lifespan2*	life span 2
			*Pbwg16*	postnatal body weight growth 16
			*Hpcr2*	hepatocarcinogen resistance 2
			*Jckm3*	juvenile cystic kidney modifier 3
			*Ath17*	atherosclerosis 17
			*Eae17*	experimental allergic encephalomyelitis susceptibility 17
			*Hfhl4*	high-frequency hearing loss 4
			*Clfrhl1*	click-frequency hearing loss 1
			*Lfhl1*	low-frequency hearing loss 1
			*Mfhl1*	medium-frequency hearing loss 1
			*Obrq12*	obesity resistance QTL 12
			*Pcholq4*	plasma cholesterol QTL 4
			*Gluq1*	blood glucose QTL 1
			*Bsc1*	brain size control 1
			*El3*	epilepsy 3
			*Insq9*	insulin QTL 9
			*Aem3*	anti-erythrocyte autoantibody modifier 3
			*Ssrq4*	stress response QTL 4
			*Pwgrq19*	post-weaning growth rate QTL 19
			*Tesq3*	testis weight QTL 3
			*Espq3*	embryo survival preimplantation QTL 3
			*Estoq3*	embryo survival total QTL 3
			*Egq9*	early growth QTL 9
			*Lgaq5*	late growth adjusted QTL 5
			*W10q8*	weight 10 weeks QTL 8
			*W6q8*	weight 6 weeks QTL 8
			*Cia29*	collagen induced arthritis QTL 29
			*Igf1sl2*	IGF-1 serum levels 2
			*Ltpr5a*	Leishmania tropica response 5a
			*Ltpr5*	Leishmania tropica response 5
			*Lgth11*	body length 11
			*Vtbt9*	vertebral trabecular bone trait 9
			*Fembrs3*	femur breaking strength 3
			*Ednrbm1*	endothelin receptor type B modifier 1
			*Tmevp2*	Theiler’s murine encephalomyelitis virus persistence 2
			*Ogrq4*	overall growth rate QTL 4
			*Femwf3*	femur work to failure 3
SB3	12		*Pifs1*	peptide-induced fatal syndrome 1
			*Tcq14*	total cholesterol QTL 14
			*Bmiq11*	body mass index QTL 11
			*Chldq7*	cholesterol and HDL QTL 7
			*Tglq5*	triglyceride QTL 5
			*Femwf9*	femur work to failure 9
			*Tmc1m3*	Tmc1 modifier 3
Total Polyps	16	*Mom 23*	*Eae11*	experimental allergic encephalomyelitis susceptibility 11
		*Mom 22*	*Arrd3*	age-related retinal degeneration 3
		*Mom 22 / Mom 23*	*Aod1a*	autoimmune ovarian dysgenesis 1a
			*Tauph*	tau phosphorylation
		*Mom 23*	*Lith14*	lithogenic gene 14
		*Mom 22*	*Remslp3*	rapid eye movement sleep 3
			*Etia*	ethanol induced activation
			*Pgia10*	proteoglycan induced arthritis 10
			*Renf2*	renal failure 2
			*Sluc27*	susceptibility to lung cancer 27
			*Bpq9*	blood pressure QTL 9
		*Mom 22*	*Pcd4ts3*	p-glycoprotein positive CD4 T cell subset 3
		*Mom 22*	*Ipng3*	imprinted postnatal growth 3
			*Tgq28*	triglyceride QTL 28
		*Mom 23*	*Imraq3*	immune response to AAV2 QTL 3
		*Mom 22 / Mom 23*	*Diobq*	diet-induced obesity QTL
		*Mom 22 / Mom 23*	*Bwtn1*	body weight at necropsy 1
		*Mom 22*	*Lp1*	lymphocyte proliferation 1
		*Mom 22 / Mom 23*	*Dice1b*	determination of interleukin 4 commitment 1b
			*Lmr18*	leishmaniasis resistance 18
		*Mom 23*	*Pod*	plasticity of ocular dominance
		*Mom 22 / Mom 23*	*Eih3*	ethanol induced hypothermia 3
		*Mom 22 / Mom 23*	*Skts-fp3*	skin tumor susceptibility in FVB and PWK 3
			*Cocia19*	cocaine-induced activity, QTL 19
			*Lmr18a*	leishmaniasis resistance 18a
			*Lmr18b*	leishmaniasis resistance 18b
		*Mom 22 / Mom 23*	*Eae41*	experimental allergic encephalomyelitis susceptibility 41
Total Polyps	12		*Ath6*	atherosclerosis 6
			*Circp1*	circadian photosensitivity 1
			*Cplaq10*	circadian period of locomotor activity 10
			*Pifs1*	peptide-induced fatal syndrome 1
			*Tcq14*	total cholesterol QTL 14
			*Bmiq11*	body mass index QTL 11
			*Chldq7*	cholesterol and HDL QTL 7
			*Tglq5*	triglyceride QTL 5
			*Femwf9*	femur work to failure 9
			*Tmc1m3*	Tmc1 modifier 3

Finally, when we combined these results with the merge analysis, we shortened the list to 123 genes (Table 5), only, while some are overlapping between the three approaches (i.e. QTL analysis at 90 and 95% genome-wide significance threshold (1862 genes), BioInfoMiner (411 genes), and Merge analysis (123 genes). These approaches have shown the power of identifying of candidate genes, which may lead to future plans for further studies with these genes.

## Discussion

Colorectal cancer is a complex disease, with many genes modifying the expression of the phenotype. The presence of mutations in the *Apc* gene alone cannot explain the wide range of different clinical features observed. It is well documented that modifying genes (host genes that modulate and regulate the expression and severity of the cancer development) have a crucial role on tumorgenesis [32]. The modifier genes of CRC have been studied in human GWAS and numerous loci found [33–36]. The combined effects of these mapped and identified alleles are currently too small to explain the bulk of heritable disease risk [33–36]. These suggest that genetic influence towards cancer susceptibility cannot be unraveled solely using approaches designed to identify the main effects of individual alleles in human populations.

Experimental mouse models are ideal for examining the effects of genetic modifiers. By crossing the mutant into a population of mice with different genetic backgrounds of naturally occurring variation, it is possible to map modifier loci. Several earlier studies [5, 29, 37, 38] on mouse models have been performed that mapped 18 *Moms*. The most tightly mapped of these QTLs (width 7.4 Mb) was found for *Mom7* [37], while the rest are between 16 to 53 Mb.

Here, we present a genetic analysis of intestinal polyp counts in 49 F1 CC-B/6-*Apc*^*Min*/+^crosses to search for *Moms*. We observed wide heritable variations in polyp counts between the 49 crosses, in accordance with our previous study that showed mice with different genetic backgrounds vary in their progression of intestinal polyp development [39]. Polyp counts in F1 CC-B/6-*Apc*^*Min*/+^crosses differ from those in the parental line (B/6-*Apc*^*Min*/+^). This suggests that the CC population contains modifiers that either suppressor enhance *Apc*^*Min*/+^ mutation, which might be caused by the high genetic diversity of the three wild-derived strains. This variation enabled us to identify new *Mom* QTL and improve the resolution of previously mapped modifiers. By using founder effect analysis, we found the haplotypic effects of founder CC stains varied between QTL and could have positive or negative effects.

The density of polyps varies across the gastrointestinal tract, suggesting tumorigenesis in different parts of the intestine is controlled by different genes and with distinct genetic architectures. Each part of the intestinal tract has specific physiological functions, with different gene expression profiles, pH and microbiota [40]. In our study we counted polyps in each of the proximal (SB1), middle (SB2) or distal (SB3) parts of small intestine and colon. Most polyps were found within the small intestine, in accordance to a previous study that showed *Apc*^*Min*/+^ mice usually develop polyps in the small intestine, unlike Familial adenomatous polyposis (FAP) patients [41]. Within the small intestine, there was no preferred location for polyp development.

We mapped nine distinct and novel *Mom* QTLs at the 90% genome-wide significance threshold, and at least an additional 16 more potential QTL at 50% threshold, but will not be fully discussed here. These QTLs are designated as modifiers of Min gene (*Mom*) numbers 19–27, respectively. We found different *Moms* to be responsible for polyp development in different parts of the intestinal tract. The wild-derived CC founder strains contain genetic variations absent from standard laboratory mouse strain (SLMS), explaining why we were able to map novel QTLs. However, in this study we were able to map some QTL associated with SLMS, as well.

Most of the *Moms* mapped in this study are distinct from those identified previously [29]. However, we mapped *Mom*27 for colon polyp development on chromosome 6:34.72–38.33 Mb, which overlap with a previously mapped *Mom*12 with wider range of 6:17.3–50.8 Mb [38]. Additionally, *Mom*26 which mapped on chromosome 9:32.56–42.56 Mb partly overlaps Colon Cancer loci susceptibility 4 (*Ccs*4) mapped to 36.84–49.23 Mb [42]. We mapped *Mom*21 to 10:8.90–28.47 Mb which does not overlap *Mom*17 (10:69–89 Mb) [8], so these probably represent distinct loci. The rest of our genome-wide significant QTLs are novel. Some of the 16 potential QTLs at 50% genome-wide significance [Table 3] overlap with previously mapped *Moms* i.e. *Mom*1 [5] and *Mom*2 [45] on chromosomes 4 and 18, respectively. |.

We have identified candidate genes underlying these traits. In this study we adopted three approaches for identifying and suggesting candidate genes (i.e. QTL analysis at 90 and 95% genome-wide significance threshold (1862 genes), BioInfoMiner (411 genes), and Merge analysis (123 genes), including list of genes identified in the genomic intervals of the mapped QTL, based on mouse genome data base, Bioinfominer analysis using Gene Ontology (GO) [26], Reactome [27] and MGI Mammalian Phenotype (MGI) data, so to identify prioritized genes with central functional and regulatory roles in enriched processes, underlying the studied phenotype, and finally based on the merge analysis. Indeed, each approach has suggested different number of genes, while the lowest number was obtained by merge analysis (123 genes).

This report and our previous study [16, 17, 39] demonstrate the utility of the CC lines in the analysis of complex traits in mouse models of human disease. This is study showed the power of using CC mice to dissect the genetic response to intestinal cancer development and the first to use of the CC F1 cross design for modifier mapping. Even a modest number of lines [16, 17] are useful with sufficient replication (3–5 mice) within each line. Similar recent studies were reported by using this CCXMutant F1 approach for defining the genetic mechanisms of host susceptibility to melanoma [43, 44].

The genomic intervals of the mapped QTL in this study were small enough to suggest candidate genes, although further confirmation work is required, including knockout or knockdown of specific candidate genes analysis. Many of these candidates are involved in innate and adaptive immune responses.

## Conclusions

Variation in polyp development is heritable and controlled, to an appreciable extent, by genetic factors segregating in the CC population which is therefore well-suited for identifying novel modifier genes associated with *Apc*^*Min/+*^mutation. The expected findings from this study may be used for early prediction of potential intestine cancer development in host carrying susceptible genetic factors, thus can be applied for better control and sufficient application therapy tools and approaches.

## Supplementary Information

**Additional file 1: Supplement 1.** Identified 411 unique genes related to the studied traits.

## Data Availability

Availability of Data: All phenotype, genotype, and bioinformatics analysis programs should be freely following DOIs: phenoPolyps.txt 10.5522/04/12790100 R codes 10.5522/04/12790160 Genotypes 10.5522/04/12790187

## Abbreviations

CEO: Chief executive officer
SME: Small medium enterprise
UK: United of Kingdom
APC: Adenomatous polyposis coli gene
Min: Multiple intestinal neoplasia
APC ^Min/+^: Mice carrying a nonsense mutation in the *Apc* gene at R850, which is designated *Apc*^*Min/*^
CRC: Colorectal cancer
MOM: modifier loci of Min
FAP: Familial Adenomatous Polyposis
Mb: Megabases
CC: Collaborative Cross
GRP: Genetic Reference Panel
HFD: High-Fat Diet
RIL: Recombinant Inbred Lines
GWAS: Genome wide association studies
QTL: Quantitative Trait Loci
TAU: Tel-Aviv University
SE: Standard Error
GO: Gene Ontology
MGI: Mouse Genome Informatics
CI: Confidence Interval
BXD: C57BL/6 JXDBA2/J mice
*M.m*: *Mus musculus*
ANOVA: Analysis of variance
SB: Small intestinal tract
GWSL: genome-wide significant thresholds levels
Go: Gene Ontology
