# Supplementary material for: Genetic mapping of novel modifiers for ApcMin induced intestinal polyps’ development using the genetic architecture power of the collaborative cross mice
Source: BMC Genomics. 2021 Jul 22;22:566. doi: 10.1186/s12864-021-07890-x (PMC8299641; doi:10.1186/s12864-021-07890-x)
Supplement: Supplementary file 1 — Additional file 1: Supplement 1. Identified 411 unique genes related to the studied traits. [file 12864_2021_7890_MOESM1_ESM.docx]

**Supplement 1. Identified 411 unique genes related to the studied traits**

| **Phenotype** | | **Number of Unique Prioritized Genes** | **Gene Names** | **Gene Descriptions** | |  |  |  |  |  |  |
| --- | --- | --- | --- | --- | --- | --- | --- | --- | --- | --- | --- |
| **SB1** | **140** | | Akt1 | thymoma viral proto-oncogene 1 | | |  |  |  |  |  |
|  |  | | Bcl11b | B cell leukemia/lymphoma 11B | | |  |  |  |  |  |
|  |  | | Dlk1 | delta like non-canonical Notch ligand 1 | | |  |  |  |  |  |
|  |  | | Vipr2 | vasoactive intestinal peptide receptor 2 | | |  |  |  |  |  |
|  |  | | Dio3 | deiodinase, iodothyronine type III | | |  |  |  |  |  |
|  |  | | Ighg1 | immunoglobulin heavy constant gamma 1 (G1m marker) | | | | |  |  |  |
|  |  | | Meg3 | maternally expressed 3 | |  |  |  |  |  |  |
|  |  | | Dync1h1 | dynein cytoplasmic 1 heavy chain 1 | | |  |  |  |  |  |
|  |  | | Sp8 | trans-acting transcription factor 8 | | |  |  |  |  |  |
|  |  | | Unc79 | unc-79 homolog | |  |  |  |  |  |  |
|  |  | | E2f5 | E2F transcription factor 5 | |  |  |  |  |  |  |
|  |  | | Gsc | goosecoid homeobox | |  |  |  |  |  |  |
|  |  | | Tdrd9 | tudor domain containing 9 | |  |  |  |  |  |  |
|  |  | | Car2 | carbonic anhydrase 2 | |  |  |  |  |  |  |
|  |  | | Jag2 | jagged 2 |  |  |  |  |  |  |  |
|  |  | | Ighv3-1 | immunoglobulin heavy variable 3-1 | | |  |  |  |  |  |
|  |  | | Ighv5-9 | immunoglobulin heavy variable 5-9 | | |  |  |  |  |  |
|  |  | | Ighv3-5 | immunoglobulin heavy variable 3-5 | | |  |  |  |  |  |
|  |  | | Ighv3-4 | immunoglobulin heavy variable V3-4 | | |  |  |  |  |  |
|  |  | | Ighv3-6 | immunoglobulin heavy variable 3-6 | | |  |  |  |  |  |
|  |  | | Ighv5-12-4 | immunoglobulin heavy variable 5-12-4 | | |  |  |  |  |  |
|  |  | | Ighv5-2 | immunoglobulin heavy variable 5-2 | | |  |  |  |  |  |
|  |  | | Ighv5-6 | immunoglobulin heavy variable 5-6 | | |  |  |  |  |  |
|  |  | | Ighv5-4 | immunoglobulin heavy variable 5-4 | | |  |  |  |  |  |
|  |  | | Ighv5-15 | immunoglobulin heavy variable 5-15 | | |  |  |  |  |  |
|  |  | | Ighv5-9-1 | immunoglobulin heavy variable 5-9-1 | | |  |  |  |  |  |
|  |  | | Ighv12-3 | immunoglobulin heavy variable V12-3 | | |  |  |  |  |  |
|  |  | | Ighv9-3 | immunoglobulin heavy variable V9-3 | | |  |  |  |  |  |
|  |  | | Ighv3-8 | immunoglobulin heavy variable V3-8 | | |  |  |  |  |  |
|  |  | | Ighv8-11 | immunoglobulin heavy variable V8-11 | | |  |  |  |  |  |
|  |  | | Ighv8-12 | immunoglobulin heavy variable V8-12 | | |  |  |  |  |  |
|  |  | | Ighv8-13 | immunoglobulin heavy variable 8-13 | | |  |  |  |  |  |
|  |  | | Ighv8-2 | immunoglobulin heavy variable V8-2 | | |  |  |  |  |  |
|  |  | | Ighv8-6 | immunoglobulin heavy variable V8-6 | | |  |  |  |  |  |
|  |  | | Ighv8-4 | immunoglobulin heavy variable V8-4 | | |  |  |  |  |  |
|  |  | | Ighv8-5 | immunoglobulin heavy variable V8-5 | | |  |  |  |  |  |
|  |  | | Ighv5-17 | immunoglobulin heavy variable 5-17 | | |  |  |  |  |  |
|  |  | | Ighv5-16 | immunoglobulin heavy variable 5-16 | | |  |  |  |  |  |
|  |  | | Ighv8-8 | immunoglobulin heavy variable 8-8 | | |  |  |  |  |  |
|  |  | | Ighv8-9 | immunoglobulin heavy variable V8-9 | | |  |  |  |  |  |
|  |  | | Ighv5-12 | immunoglobulin heavy variable 5-12 | | |  |  |  |  |  |
|  |  | | Ighv9-2 | immunoglobulin heavy variable V9-2 | | |  |  |  |  |  |
|  |  | | Ighv9-4 | immunoglobulin heavy variable 9-4 | | |  |  |  |  |  |
|  |  | | Ighv9-1 | immunoglobulin heavy variable 9-1 | | |  |  |  |  |  |
|  |  | | Ighv3-3 | immunoglobulin heavy variable V3-3 | | |  |  |  |  |  |
|  |  | | Ighg2c | immunoglobulin heavy constant gamma 2C | | | |  |  |  |  |
|  |  | | Ighg2b | immunoglobulin heavy constant gamma 2B | | | |  |  |  |  |
|  |  | | Ighg3 | Immunoglobulin heavy constant gamma 3 | | | |  |  |  |  |
|  |  | | Ighv15-2 | immunoglobulin heavy variable V15-2 | | |  |  |  |  |  |
|  |  | | Ighv2-6-8 | immunoglobulin heavy variable 2-6-8 | | |  |  |  |  |  |
|  |  | | Ighv1-26 | immunoglobulin heavy variable 1-26 | | |  |  |  |  |  |
|  |  | | Ighv1-24 | immunoglobulin heavy variable V1-24 | | |  |  |  |  |  |
|  |  | | Ighv1-22 | immunoglobulin heavy variable 1-22 | | |  |  |  |  |  |
|  |  | | Ighv16-1 | immunoglobulin heavy variable 16-1 | | |  |  |  |  |  |
|  |  | | Ighv1-20 | immunoglobulin heavy variable V1-20 | | |  |  |  |  |  |
|  |  | | Ighv11-2 | immunoglobulin heavy variable V11-2 | | |  |  |  |  |  |
|  |  | | Ighv11-1 | immunoglobulin heavy variable 11-1 | | |  |  |  |  |  |
|  |  | | Ighv1-39 | immunoglobulin heavy variable 1-39 | | |  |  |  |  |  |
|  |  | | Ighv1-31 | immunoglobulin heavy variable 1-31 | | |  |  |  |  |  |
|  |  | | Ighv1-34 | immunoglobulin heavy variable 1-34 | | |  |  |  |  |  |
|  |  | | Ighv1-37 | immunoglobulin heavy variable 1-37 | | |  |  |  |  |  |
|  |  | | Ighv1-36 | immunoglobulin heavy variable 1-36 | | |  |  |  |  |  |
|  |  | | Ighv2-9-1 | immunoglobulin heavy variable 2-9-1 | | |  |  |  |  |  |
|  |  | | Ighv1-9 | immunoglobulin heavy variable V1-9 | | |  |  |  |  |  |
|  |  | | Ighv1-7 | immunoglobulin heavy variable V1-7 | | |  |  |  |  |  |
|  |  | | Ighv1-5 | immunoglobulin heavy variable V1-5 | | |  |  |  |  |  |
|  |  | | Ighv1-4 | immunoglobulin heavy variable 1-4 | | |  |  |  |  |  |
|  |  | | Ighv1-49 | immunoglobulin heavy variable 1-49 | | |  |  |  |  |  |
|  |  | | Ighv13-2 | immunoglobulin heavy variable 13-2 | | |  |  |  |  |  |
|  |  | | Ighv1-47 | immunoglobulin heavy variable 1-47 | | |  |  |  |  |  |
|  |  | | Ighv1-42 | immunoglobulin heavy variable V1-42 | | |  |  |  |  |  |
|  |  | | Ighv1-43 | immunoglobulin heavy variable V1-43 | | |  |  |  |  |  |
|  |  | | Igha | immunoglobulin heavy constant alpha | | |  |  |  |  |  |
|  |  | | Ighd | immunoglobulin heavy constant delta | | |  |  |  |  |  |
|  |  | | Ighv1-59 | immunoglobulin heavy variable V1-59 | | |  |  |  |  |  |
|  |  | | Ighv1-58 | immunoglobulin heavy variable 1-58 | | |  |  |  |  |  |
|  |  | | Ighv1-56 | immunoglobulin heavy variable 1-56 | | |  |  |  |  |  |
|  |  | | Ighv1-55 | immunoglobulin heavy variable 1-55 | | |  |  |  |  |  |
|  |  | | Ighv1-54 | immunoglobulin heavy variable V1-54 | | |  |  |  |  |  |
|  |  | | Ighv1-53 | immunoglobulin heavy variable 1-53 | | |  |  |  |  |  |
|  |  | | Ighv1-52 | immunoglobulin heavy variable 1-52 | | |  |  |  |  |  |
|  |  | | Ighv1-23 | immunoglobulin heavy variable V1-23 | | |  |  |  |  |  |
|  |  | | Ighv4-1 | immunoglobulin heavy variable 4-1 | | |  |  |  |  |  |
|  |  | | Ighv7-4 | immunoglobulin heavy variable 7-4 | | |  |  |  |  |  |
|  |  | | Ighv7-1 | immunoglobulin heavy variable 7-1 | | |  |  |  |  |  |
|  |  | | Ighv7-3 | immunoglobulin heavy variable 7-3 | | |  |  |  |  |  |
|  |  | | Ighv14-2 | immunoglobulin heavy variable 14-2 | | |  |  |  |  |  |
|  |  | | Ighv14-3 | immunoglobulin heavy variable V14-3 | | |  |  |  |  |  |
|  |  | | Ighv14-1 | immunoglobulin heavy variable 14-1 | | |  |  |  |  |  |
|  |  | | Ighv14-4 | immunoglobulin heavy variable 14-4 | | |  |  |  |  |  |
|  |  | | Ighv1-63 | immunoglobulin heavy variable V1-63 | | |  |  |  |  |  |
|  |  | | Ighv1-61 | immunoglobulin heavy variable 1-61 | | |  |  |  |  |  |
|  |  | | Ighv1-66 | immunoglobulin heavy variable 1-66 | | |  |  |  |  |  |
|  |  | | Ighv1-67 | immunoglobulin heavy variable V1-67 | | |  |  |  |  |  |
|  |  | | Ighv1-64 | immunoglobulin heavy variable 1-64 | | |  |  |  |  |  |
|  |  | | Ighv1-69 | immunoglobulin heavy variable 1-69 | | |  |  |  |  |  |
|  |  | | Ighv10-3 | immunoglobulin heavy variable V10-3 | | |  |  |  |  |  |
|  |  | | Ighv10-1 | immunoglobulin heavy variable 10-1 | | |  |  |  |  |  |
|  |  | | Ighv1-50 | immunoglobulin heavy variable 1-50 | | |  |  |  |  |  |
|  |  | | Ighv2-9 | immunoglobulin heavy variable 2-9 | | |  |  |  |  |  |
|  |  | | Ighv1-75 | immunoglobulin heavy variable 1-75 | | |  |  |  |  |  |
|  |  | | Ighv1-77 | immunoglobulin heavy variable 1-77 | | |  |  |  |  |  |
|  |  | | Ighv1-76 | immunoglobulin heavy variable 1-76 | | |  |  |  |  |  |
|  |  | | Ighv1-71 | immunoglobulin heavy variable 1-71 | | |  |  |  |  |  |
|  |  | | Ighv1-62-3 | immunoglobulin heavy variable 1-62-3 | | |  |  |  |  |  |
|  |  | | Ighv1-62-2 | immunoglobulin heavy variable 1-62-2 | | |  |  |  |  |  |
|  |  | | Ighv6-4 | immunoglobulin heavy variable V6-4 | | |  |  |  |  |  |
|  |  | | Ighv1-78 | immunoglobulin heavy variable 1-78 | | |  |  |  |  |  |
|  |  | | Ighv6-5 | immunoglobulin heavy variable V6-5 | | |  |  |  |  |  |
|  |  | | Ighv6-6 | immunoglobulin heavy variable 6-6 | | |  |  |  |  |  |
|  |  | | Ighv2-5 | immunoglobulin heavy variable 2-5 | | |  |  |  |  |  |
|  |  | | Ighv2-6 | immunoglobulin heavy variable 2-6 | | |  |  |  |  |  |
|  |  | | Ighv2-7 | immunoglobulin heavy variable 2-7 | | |  |  |  |  |  |
|  |  | | Ighv2-2 | immunoglobulin heavy variable 2-2 | | |  |  |  |  |  |
|  |  | | Ighv2-3 | immunoglobulin heavy variable 2-3 | | |  |  |  |  |  |
|  |  | | Ighv7-2 | immunoglobulin heavy variable 7-2 | | |  |  |  |  |  |
|  |  | | Ighv1-80 | immunoglobulin heavy variable 1-80 | | |  |  |  |  |  |
|  |  | | Ighv1-81 | immunoglobulin heavy variable 1-81 | | |  |  |  |  |  |
|  |  | | Ighv1-82 | immunoglobulin heavy variable 1-82 | | |  |  |  |  |  |
|  |  | | Ighv1-85 | immunoglobulin heavy variable 1-85 | | |  |  |  |  |  |
|  |  | | Ighv1-12 | immunoglobulin heavy variable V1-12 | | |  |  |  |  |  |
|  |  | | Ighv1-11 | immunoglobulin heavy variable V1-11 | | |  |  |  |  |  |
|  |  | | Ighv6-3 | immunoglobulin heavy variable 6-3 | | |  |  |  |  |  |
|  |  | | Ighv1-16 | immunoglobulin heavy variable 1-16 | | |  |  |  |  |  |
|  |  | | Ighv1-15 | immunoglobulin heavy variable 1-15 | | |  |  |  |  |  |
|  |  | | Ighv6-7 | immunoglobulin heavy variable V6-7 | | |  |  |  |  |  |
|  |  | | Ighv1-19 | immunoglobulin heavy variable V1-19 | | |  |  |  |  |  |
|  |  | | Ighv1-18 | immunoglobulin heavy variable V1-18 | | |  |  |  |  |  |
|  |  | | Ighv1-62-1 | immunoglobulin heavy variable 1-62-1 | | |  |  |  |  |  |
|  |  | | Chga | chromogranin A | |  |  |  |  |  |  |
|  |  | | Siva1 | SIVA1, apoptosis-inducing factor | | |  |  |  |  |  |
|  |  | | Moap1 | modulator of apoptosis 1 | |  |  |  |  |  |  |
|  |  | | Ighm | immunoglobulin heavy constant mu | | |  |  |  |  |  |
|  |  | | Traf3 | TNF receptor-associated factor 3 | | |  |  |  |  |  |
|  |  | | Hsp90aa1 | heat shock protein 90, alpha (cytosolic), class A member 1 | | | | |  |  |  |
|  |  | | Pld4 | phospholipase D family, member 4 | | |  |  |  |  |  |
|  |  | | Bdkrb2 | bradykinin receptor, beta 2 | | |  |  |  |  |  |
|  |  | | Bdkrb1 | bradykinin receptor, beta 1 | | |  |  |  |  |  |
|  |  | | Fabp4 | fatty acid binding protein 4, adipocyte | | |  |  |  |  |  |
|  |  | | Cyp7b1 | cytochrome P450, family 7, subfamily b, polypeptide 1 | | | | |  |  |  |
|  |  | |  |  |  |  |  |  |  |  |  |
| **SB2** | **56** | | Arg1 | arginase, liver | |  |  |  |  |  |  |
|  |  | | H60b | histocompatibility 60b | |  |  |  |  |  |  |
|  |  | | Raet1e | retinoic acid early transcript 1E | | |  |  |  |  |  |
|  |  | | Raet1d | retinoic acid early transcript delta | | |  |  |  |  |  |
|  |  | | Tnfaip3 | tumor necrosis factor, alpha-induced protein 3 | | | |  |  |  |  |
|  |  | | Myb | myeloblastosis oncogene | |  |  |  |  |  |  |
|  |  | | Cited2 | Cbp/p300-interacting transactivator, with Glu/Asp-rich | | | | | | |  |
|  |  | | Cd96 | CD96 antigen | |  |  |  |  |  |  |
|  |  | | Cblb | Casitas B-lineage lymphoma b | | |  |  |  |  |  |
|  |  | | Samsn1 | SAM domain, SH3 domain and nuclear localization signals, 1 | | | | |  |  |  |
|  |  | | Enpp3 | ectonucleotide pyrophosphatase/phosphodiesterase 3 | | | | |  |  |  |
|  |  | | Robo2 | roundabout guidance receptor 2 | | |  |  |  |  |  |
|  |  | | Map3k5 | mitogen-activated protein kinase kinase kinase 5 | | | |  |  |  |  |
|  |  | | Eya4 | EYA transcriptional coactivator and phosphatase 4 | | | |  |  |  |  |
|  |  | | Robo1 | roundabout guidance receptor 1 | | |  |  |  |  |  |
|  |  | | Phldb2 | pleckstrin homology like domain, family B, member 2 | | | |  |  |  |  |
|  |  | | Nfkbiz | nuclear factor of kappa light polypeptide gene enhancer in B cells | | | | | |  |  |
|  |  | | Vnn1 | vanin 1 |  |  |  |  |  |  |  |
|  |  | | Cd47 | CD47 antigen (Rh-related antigen, integrin-associated signal | | | | | |  |  |
|  |  | | Stx7 | syntaxin 7 |  |  |  |  |  |  |  |
|  |  | | Fuca2 | fucosidase, alpha-L- 2, plasma | | |  |  |  |  |  |
|  |  | | E330017A01Rik | RIKEN cDNA E330017A01 gene | | |  |  |  |  |  |
|  |  | | Taar5 | trace amine-associated receptor 5 | | |  |  |  |  |  |
|  |  | | Taar6 | trace amine-associated receptor 6 | | |  |  |  |  |  |
|  |  | | Taar1 | trace amine-associated receptor 1 | | |  |  |  |  |  |
|  |  | | Taar3 | trace amine-associated receptor 3 | | |  |  |  |  |  |
|  |  | | Taar2 | trace amine-associated receptor 2 | | |  |  |  |  |  |
|  |  | | Taar9 | trace amine-associated receptor 9 | | |  |  |  |  |  |
|  |  | | Taar8b | trace amine-associated receptor 8B | | |  |  |  |  |  |
|  |  | | Olfr196 | olfactory receptor 196 | |  |  |  |  |  |  |
|  |  | | Nmbr | neuromedin B receptor | |  |  |  |  |  |  |
|  |  | | Olfr193 | olfactory receptor 193 | |  |  |  |  |  |  |
|  |  | | Olfr191 | olfactory receptor 191 | |  |  |  |  |  |  |
|  |  | | Olfr198 | olfactory receptor 198 | |  |  |  |  |  |  |
|  |  | | Olfr203 | olfactory receptor 203 | |  |  |  |  |  |  |
|  |  | | Olfr172 | olfactory receptor 172 | |  |  |  |  |  |  |
|  |  | | Olfr173 | olfactory receptor 173 | |  |  |  |  |  |  |
|  |  | | Olfr183 | olfactory receptor 183 | |  |  |  |  |  |  |
|  |  | | Olfr187 | olfactory receptor 187 | |  |  |  |  |  |  |
|  |  | | Olfr186 | olfactory receptor 186 | |  |  |  |  |  |  |
|  |  | | Olfr195 | olfactory receptor 195 | |  |  |  |  |  |  |
|  |  | | Gpr15 | G protein-coupled receptor 15 | | |  |  |  |  |  |
|  |  | | Pde7b | phosphodiesterase 7B | |  |  |  |  |  |  |
|  |  | | Grm1 | glutamate receptor, metabotropic 1 | | |  |  |  |  |  |
|  |  | | Lama2 | laminin, alpha 2 | |  |  |  |  |  |  |
|  |  | | Ifngr1 | interferon gamma receptor 1 | | |  |  |  |  |  |
|  |  | | Retnla | resistin like alpha | |  |  |  |  |  |  |
|  |  | | Epm2a | epilepsy, progressive myoclonic epilepsy, type 2 gene alpha | | | | |  |  |  |
|  |  | | Hbs1l | Hbs1-like (S. cerevisiae) | |  |  |  |  |  |  |
|  |  | | Pex7 | peroxisomal biogenesis factor 7 | | |  |  |  |  |  |
|  |  | | Enpp1 | ectonucleotide pyrophosphatase/phosphodiesterase 1 | | | | |  |  |  |
|  |  | | Retnlb | resistin like beta | |  |  |  |  |  |  |
|  |  | | Usp25 | ubiquitin specific peptidase 25 | | |  |  |  |  |  |
|  |  | | Morc1 | microrchidia 1 | |  |  |  |  |  |  |
|  |  | | Sgk1 | serum/glucocorticoid regulated kinase 1 | | | |  |  |  |  |
|  |  | | Pros1 | protein S (alpha) | |  |  |  |  |  |  |
|  |  | |  |  |  |  |  |  |  |  |  |
| **SB3** | **150** | | Epm2a | epilepsy, progressive myoclonic epilepsy, type 2 gene alpha | | | | |  |  |  |
|  |  | | Kras | Kirsten rat sarcoma viral oncogene homolog | | | |  |  |  |  |
|  |  | | Gys2 | glycogen synthase 2 | |  |  |  |  |  |  |
|  |  | | Vipr2 | vasoactive intestinal peptide receptor 2 | | |  |  |  |  |  |
|  |  | | Pex7 | peroxisomal biogenesis factor 7 | | |  |  |  |  |  |
|  |  | | Enpp1 | ectonucleotide pyrophosphatase/phosphodiesterase 1 | | | | |  |  |  |
|  |  | | Kcnj8 | potassium inwardly-rectifying channel, subfamily J, member 8 | | | | |  |  |  |
|  |  | | Enpp3 | ectonucleotide pyrophosphatase/phosphodiesterase 3 | | | | |  |  |  |
|  |  | | Tmem218 | transmembrane protein 218 | | |  |  |  |  |  |
|  |  | | Tbrg1 | transforming growth factor beta regulated gene 1 | | | |  |  |  |  |
|  |  | | Ckb | creatine kinase, brain | |  |  |  |  |  |  |
|  |  | | Igha | immunoglobulin heavy constant alpha | | |  |  |  |  |  |
|  |  | | Ighm | immunoglobulin heavy constant mu | | |  |  |  |  |  |
|  |  | | Ifngr1 | interferon gamma receptor 1 | | |  |  |  |  |  |
|  |  | | Mark3 | MAP/microtubule affinity regulating kinase 3 | | | |  |  |  |  |
|  |  | | Hbs1l | Hbs1-like (S. cerevisiae) | |  |  |  |  |  |  |
|  |  | | Lama2 | laminin, alpha 2 | |  |  |  |  |  |  |
|  |  | | Slco1b2 | solute carrier organic anion transporter family, member 1b2 | | | | |  |  |  |
|  |  | | Dio3 | deiodinase, iodothyronine type III | | |  |  |  |  |  |
|  |  | | Jag2 | jagged 2 |  |  |  |  |  |  |  |
|  |  | | Klc1 | kinesin light chain 1 | |  |  |  |  |  |  |
|  |  | | Myb | myeloblastosis oncogene | |  |  |  |  |  |  |
|  |  | | Ighv3-1 | immunoglobulin heavy variable 3-1 | | |  |  |  |  |  |
|  |  | | Ighv5-9 | immunoglobulin heavy variable 5-9 | | |  |  |  |  |  |
|  |  | | Ighv3-5 | immunoglobulin heavy variable 3-5 | | |  |  |  |  |  |
|  |  | | Ighv3-4 | immunoglobulin heavy variable V3-4 | | |  |  |  |  |  |
|  |  | | Ighv3-6 | immunoglobulin heavy variable 3-6 | | |  |  |  |  |  |
|  |  | | Ighv5-12-4 | immunoglobulin heavy variable 5-12-4 | | |  |  |  |  |  |
|  |  | | Ighv5-2 | immunoglobulin heavy variable 5-2 | | |  |  |  |  |  |
|  |  | | Ighv5-6 | immunoglobulin heavy variable 5-6 | | |  |  |  |  |  |
|  |  | | Ighv5-4 | immunoglobulin heavy variable 5-4 | | |  |  |  |  |  |
|  |  | | Ighv5-15 | immunoglobulin heavy variable 5-15 | | |  |  |  |  |  |
|  |  | | Ighv5-9-1 | immunoglobulin heavy variable 5-9-1 | | |  |  |  |  |  |
|  |  | | Ighv12-3 | immunoglobulin heavy variable V12-3 | | |  |  |  |  |  |
|  |  | | Ighv3-8 | immunoglobulin heavy variable V3-8 | | |  |  |  |  |  |
|  |  | | Ighv8-11 | immunoglobulin heavy variable V8-11 | | |  |  |  |  |  |
|  |  | | Ighv8-12 | immunoglobulin heavy variable V8-12 | | |  |  |  |  |  |
|  |  | | Ighv8-13 | immunoglobulin heavy variable 8-13 | | |  |  |  |  |  |
|  |  | | Ighv8-2 | immunoglobulin heavy variable V8-2 | | |  |  |  |  |  |
|  |  | | Ighv8-6 | immunoglobulin heavy variable V8-6 | | |  |  |  |  |  |
|  |  | | Ighv8-4 | immunoglobulin heavy variable V8-4 | | |  |  |  |  |  |
|  |  | | Ighv8-5 | immunoglobulin heavy variable V8-5 | | |  |  |  |  |  |
|  |  | | Ighv5-17 | immunoglobulin heavy variable 5-17 | | |  |  |  |  |  |
|  |  | | Ighv5-16 | immunoglobulin heavy variable 5-16 | | |  |  |  |  |  |
|  |  | | Ighv8-8 | immunoglobulin heavy variable 8-8 | | |  |  |  |  |  |
|  |  | | Ighv8-9 | immunoglobulin heavy variable V8-9 | | |  |  |  |  |  |
|  |  | | Ighv5-12 | immunoglobulin heavy variable 5-12 | | |  |  |  |  |  |
|  |  | | Ighv9-4 | immunoglobulin heavy variable 9-4 | | |  |  |  |  |  |
|  |  | | Ighv9-3 | immunoglobulin heavy variable V9-3 | | |  |  |  |  |  |
|  |  | | Ighv9-2 | immunoglobulin heavy variable V9-2 | | |  |  |  |  |  |
|  |  | | Ighv9-1 | immunoglobulin heavy variable 9-1 | | |  |  |  |  |  |
|  |  | | Ighv3-3 | immunoglobulin heavy variable V3-3 | | |  |  |  |  |  |
|  |  | | Ighg2c | immunoglobulin heavy constant gamma 2C | | | |  |  |  |  |
|  |  | | Ighg2b | immunoglobulin heavy constant gamma 2B | | | |  |  |  |  |
|  |  | | Ighg1 | immunoglobulin heavy constant gamma 1 (G1m marker) | | | | |  |  |  |
|  |  | | Ighg3 | Immunoglobulin heavy constant gamma 3 | | | |  |  |  |  |
|  |  | | Ighv15-2 | immunoglobulin heavy variable V15-2 | | |  |  |  |  |  |
|  |  | | Ighv2-6-8 | immunoglobulin heavy variable 2-6-8 | | |  |  |  |  |  |
|  |  | | Ighv1-26 | immunoglobulin heavy variable 1-26 | | |  |  |  |  |  |
|  |  | | Ighv1-24 | immunoglobulin heavy variable V1-24 | | |  |  |  |  |  |
|  |  | | Ighv1-22 | immunoglobulin heavy variable 1-22 | | |  |  |  |  |  |
|  |  | | Ighv1-23 | immunoglobulin heavy variable V1-23 | | |  |  |  |  |  |
|  |  | | Ighv1-20 | immunoglobulin heavy variable V1-20 | | |  |  |  |  |  |
|  |  | | Ighv11-2 | immunoglobulin heavy variable V11-2 | | |  |  |  |  |  |
|  |  | | Ighv11-1 | immunoglobulin heavy variable 11-1 | | |  |  |  |  |  |
|  |  | | Ighv1-39 | immunoglobulin heavy variable 1-39 | | |  |  |  |  |  |
|  |  | | Ighv1-31 | immunoglobulin heavy variable 1-31 | | |  |  |  |  |  |
|  |  | | Ighv1-34 | immunoglobulin heavy variable 1-34 | | |  |  |  |  |  |
|  |  | | Ighv1-37 | immunoglobulin heavy variable 1-37 | | |  |  |  |  |  |
|  |  | | Ighv1-36 | immunoglobulin heavy variable 1-36 | | |  |  |  |  |  |
|  |  | | Ighv2-9-1 | immunoglobulin heavy variable 2-9-1 | | |  |  |  |  |  |
|  |  | | Ighv1-9 | immunoglobulin heavy variable V1-9 | | |  |  |  |  |  |
|  |  | | Ighv1-7 | immunoglobulin heavy variable V1-7 | | |  |  |  |  |  |
|  |  | | Ighv1-5 | immunoglobulin heavy variable V1-5 | | |  |  |  |  |  |
|  |  | | Ighv1-4 | immunoglobulin heavy variable 1-4 | | |  |  |  |  |  |
|  |  | | Ighv1-49 | immunoglobulin heavy variable 1-49 | | |  |  |  |  |  |
|  |  | | Ighv13-2 | immunoglobulin heavy variable 13-2 | | |  |  |  |  |  |
|  |  | | Ighv1-47 | immunoglobulin heavy variable 1-47 | | |  |  |  |  |  |
|  |  | | Ighv1-42 | immunoglobulin heavy variable V1-42 | | |  |  |  |  |  |
|  |  | | Ighv1-43 | immunoglobulin heavy variable V1-43 | | |  |  |  |  |  |
|  |  | | Ighd | immunoglobulin heavy constant delta | | |  |  |  |  |  |
|  |  | | Ighv1-59 | immunoglobulin heavy variable V1-59 | | |  |  |  |  |  |
|  |  | | Ighv1-58 | immunoglobulin heavy variable 1-58 | | |  |  |  |  |  |
|  |  | | Ighv1-56 | immunoglobulin heavy variable 1-56 | | |  |  |  |  |  |
|  |  | | Ighv1-55 | immunoglobulin heavy variable 1-55 | | |  |  |  |  |  |
|  |  | | Ighv1-54 | immunoglobulin heavy variable V1-54 | | |  |  |  |  |  |
|  |  | | Ighv1-53 | immunoglobulin heavy variable 1-53 | | |  |  |  |  |  |
|  |  | | Ighv1-52 | immunoglobulin heavy variable 1-52 | | |  |  |  |  |  |
|  |  | | Ighv16-1 | immunoglobulin heavy variable 16-1 | | |  |  |  |  |  |
|  |  | | Ighv4-1 | immunoglobulin heavy variable 4-1 | | |  |  |  |  |  |
|  |  | | Ighv7-4 | immunoglobulin heavy variable 7-4 | | |  |  |  |  |  |
|  |  | | Ighv7-1 | immunoglobulin heavy variable 7-1 | | |  |  |  |  |  |
|  |  | | Ighv7-3 | immunoglobulin heavy variable 7-3 | | |  |  |  |  |  |
|  |  | | Ighv14-2 | immunoglobulin heavy variable 14-2 | | |  |  |  |  |  |
|  |  | | Ighv14-3 | immunoglobulin heavy variable V14-3 | | |  |  |  |  |  |
|  |  | | Ighv14-1 | immunoglobulin heavy variable 14-1 | | |  |  |  |  |  |
|  |  | | Ighv14-4 | immunoglobulin heavy variable 14-4 | | |  |  |  |  |  |
|  |  | | Ighv2-6 | immunoglobulin heavy variable 2-6 | | |  |  |  |  |  |
|  |  | | Ighv1-63 | immunoglobulin heavy variable V1-63 | | |  |  |  |  |  |
|  |  | | Ighv1-61 | immunoglobulin heavy variable 1-61 | | |  |  |  |  |  |
|  |  | | Ighv1-66 | immunoglobulin heavy variable 1-66 | | |  |  |  |  |  |
|  |  | | Ighv1-67 | immunoglobulin heavy variable V1-67 | | |  |  |  |  |  |
|  |  | | Ighv1-64 | immunoglobulin heavy variable 1-64 | | |  |  |  |  |  |
|  |  | | Ighv1-69 | immunoglobulin heavy variable 1-69 | | |  |  |  |  |  |
|  |  | | Ighv10-3 | immunoglobulin heavy variable V10-3 | | |  |  |  |  |  |
|  |  | | Ighv10-1 | immunoglobulin heavy variable 10-1 | | |  |  |  |  |  |
|  |  | | Ighv1-50 | immunoglobulin heavy variable 1-50 | | |  |  |  |  |  |
|  |  | | Ighv2-9 | immunoglobulin heavy variable 2-9 | | |  |  |  |  |  |
|  |  | | Ighv1-75 | immunoglobulin heavy variable 1-75 | | |  |  |  |  |  |
|  |  | | Ighv1-77 | immunoglobulin heavy variable 1-77 | | |  |  |  |  |  |
|  |  | | Ighv1-76 | immunoglobulin heavy variable 1-76 | | |  |  |  |  |  |
|  |  | | Ighv1-62-1 | immunoglobulin heavy variable 1-62-1 | | |  |  |  |  |  |
|  |  | | Ighv1-62-3 | immunoglobulin heavy variable 1-62-3 | | |  |  |  |  |  |
|  |  | | Ighv1-62-2 | immunoglobulin heavy variable 1-62-2 | | |  |  |  |  |  |
|  |  | | Ighv1-78 | immunoglobulin heavy variable 1-78 | | |  |  |  |  |  |
|  |  | | Ighv6-5 | immunoglobulin heavy variable V6-5 | | |  |  |  |  |  |
|  |  | | Ighv6-6 | immunoglobulin heavy variable 6-6 | | |  |  |  |  |  |
|  |  | | Ighv2-5 | immunoglobulin heavy variable 2-5 | | |  |  |  |  |  |
|  |  | | Ighv2-7 | immunoglobulin heavy variable 2-7 | | |  |  |  |  |  |
|  |  | | Ighv2-2 | immunoglobulin heavy variable 2-2 | | |  |  |  |  |  |
|  |  | | Ighv2-3 | immunoglobulin heavy variable 2-3 | | |  |  |  |  |  |
|  |  | | Ighv7-2 | immunoglobulin heavy variable 7-2 | | |  |  |  |  |  |
|  |  | | Ighv1-80 | immunoglobulin heavy variable 1-80 | | |  |  |  |  |  |
|  |  | | Ighv1-81 | immunoglobulin heavy variable 1-81 | | |  |  |  |  |  |
|  |  | | Ighv1-82 | immunoglobulin heavy variable 1-82 | | |  |  |  |  |  |
|  |  | | Ighv1-85 | immunoglobulin heavy variable 1-85 | | |  |  |  |  |  |
|  |  | | Ighv1-12 | immunoglobulin heavy variable V1-12 | | |  |  |  |  |  |
|  |  | | Ighv1-11 | immunoglobulin heavy variable V1-11 | | |  |  |  |  |  |
|  |  | | Ighv6-3 | immunoglobulin heavy variable 6-3 | | |  |  |  |  |  |
|  |  | | Ighv6-4 | immunoglobulin heavy variable V6-4 | | |  |  |  |  |  |
|  |  | | Ighv1-16 | immunoglobulin heavy variable 1-16 | | |  |  |  |  |  |
|  |  | | Ighv1-15 | immunoglobulin heavy variable 1-15 | | |  |  |  |  |  |
|  |  | | Ighv6-7 | immunoglobulin heavy variable V6-7 | | |  |  |  |  |  |
|  |  | | Ighv1-19 | immunoglobulin heavy variable V1-19 | | |  |  |  |  |  |
|  |  | | Ighv1-18 | immunoglobulin heavy variable V1-18 | | |  |  |  |  |  |
|  |  | | Ighv1-71 | immunoglobulin heavy variable 1-71 | | |  |  |  |  |  |
|  |  | | Crtam | cytotoxic and regulatory T cell molecule | | |  |  |  |  |  |
|  |  | | Tnfaip3 | tumor necrosis factor, alpha-induced protein 3 | | | |  |  |  |  |
|  |  | | Akt1 | thymoma viral proto-oncogene 1 | | |  |  |  |  |  |
|  |  | | Traf3 | TNF receptor-associated factor 3 | | |  |  |  |  |  |
|  |  | | Siva1 | SIVA1, apoptosis-inducing factor | | |  |  |  |  |  |
|  |  | | Tirap | toll-interleukin 1 receptor (TIR) domain-containing adaptor protein | | | | |  |  |  |
|  |  | | Arg1 | arginase, liver | |  |  |  |  |  |  |
|  |  | | Raet1e | retinoic acid early transcript 1E | | |  |  |  |  |  |
|  |  | | Hspa8 | heat shock protein 8 | |  |  |  |  |  |  |
|  |  | | H60b | histocompatibility 60b | |  |  |  |  |  |  |
|  |  | | Raet1d | retinoic acid early transcript delta | | |  |  |  |  |  |
|  |  | | Abcc9 | ATP-binding cassette, sub-family C (CFTR/MRP), member 9 | | | | |  |  |  |
|  |  | | Ets1 | E26 avian leukemia oncogene 1, 5' domain | | | |  |  |  |  |
|  |  | | Cited2 | Cbp/p300-interacting transactivator, with Glu/Asp-rich | | | | | | |  |
|  |  | |  |  |  |  |  |  |  |  |  |
| **Colon** | **12** | | Ptn | pleiotrophin |  |  |  |  |  |  |  |
|  |  | | Ttc26 | tetratricopeptide repeat domain 26 | | |  |  |  |  |  |
|  |  | | Wdr91 | WD repeat domain 91 | |  |  |  |  |  |  |
|  |  | | Chrm2 | cholinergic receptor, muscarinic 2, cardiac | | | |  |  |  |  |
|  |  | | Atp6v0a4 | ATPase, H+ transporting, lysosomal V0 subunit A4 | | | |  |  |  |  |
|  |  | | Mtpn | myotrophin |  |  |  |  |  |  |  |
|  |  | | Nup205 | nucleoporin 205 | |  |  |  |  |  |  |
|  |  | | Creb3l2 | cAMP responsive element binding protein 3-like 2 | | | |  |  |  |  |
|  |  | | Slc13a4 | solute carrier family 13 (sodium/sulfate symporters), member 4 | | | | |  |  |  |
|  |  | | Cnot4 | CCR4-NOT transcription complex, subunit 4 | | | |  |  |  |  |
|  |  | | Dgki | diacylglycerol kinase, iota | |  |  |  |  |  |  |
|  |  | | Akr1d1 | aldo-keto reductase family 1, member D1 | | | |  |  |  |  |
|  |  | |  |  |  |  |  |  |  |  |  |
| **Total Polyps** | **53** | | Robo1 | roundabout guidance receptor 1 | | |  |  |  |  |  |
|  |  | | Btla | B and T lymphocyte associated | | |  |  |  |  |  |
|  |  | | Ighm | immunoglobulin heavy constant mu | | |  |  |  |  |  |
|  |  | | Robo2 | roundabout guidance receptor 2 | | |  |  |  |  |  |
|  |  | | Akt1 | thymoma viral proto-oncogene 1 | | |  |  |  |  |  |
|  |  | | Retnlb | resistin like beta | |  |  |  |  |  |  |
|  |  | | Retnla | resistin like alpha | |  |  |  |  |  |  |
|  |  | | Bbx | bobby sox HMG box containing | | |  |  |  |  |  |
|  |  | | Usp25 | ubiquitin specific peptidase 25 | | |  |  |  |  |  |
|  |  | | Igha | immunoglobulin heavy constant alpha | | |  |  |  |  |  |
|  |  | | Nfkbiz | nuclear factor of kappa light polypeptide gene enhancer in B cells | | | | | |  |  |
|  |  | | Ighd | immunoglobulin heavy constant delta | | |  |  |  |  |  |
|  |  | | Ighe | Immunoglobulin heavy constant epsilon | | |  |  |  |  |  |
|  |  | | Gpr132 | G protein-coupled receptor 132 | | |  |  |  |  |  |
|  |  | | Cd200 | CD200 antigen | |  |  |  |  |  |  |
|  |  | | Cxadr | coxsackie virus and adenovirus receptor | | |  |  |  |  |  |
|  |  | | Cblb | Casitas B-lineage lymphoma b | | |  |  |  |  |  |
|  |  | | Ighg1 | immunoglobulin heavy constant gamma 1 (G1m marker) | | | | |  |  |  |
|  |  | | Ighv5-9 | immunoglobulin heavy variable 5-9 | | |  |  |  |  |  |
|  |  | | Ighv5-12-4 | immunoglobulin heavy variable 5-12-4 | | |  |  |  |  |  |
|  |  | | Ighv5-2 | immunoglobulin heavy variable 5-2 | | |  |  |  |  |  |
|  |  | | Ighv5-6 | immunoglobulin heavy variable 5-6 | | |  |  |  |  |  |
|  |  | | Ighv5-4 | immunoglobulin heavy variable 5-4 | | |  |  |  |  |  |
|  |  | | Ighv5-17 | immunoglobulin heavy variable 5-17 | | |  |  |  |  |  |
|  |  | | Ighv5-16 | immunoglobulin heavy variable 5-16 | | |  |  |  |  |  |
|  |  | | Ighv5-15 | immunoglobulin heavy variable 5-15 | | |  |  |  |  |  |
|  |  | | Ighv5-12 | immunoglobulin heavy variable 5-12 | | |  |  |  |  |  |
|  |  | | Ighv5-9-1 | immunoglobulin heavy variable 5-9-1 | | |  |  |  |  |  |
|  |  | | Ighg2c | immunoglobulin heavy constant gamma 2C | | | |  |  |  |  |
|  |  | | Ighg2b | immunoglobulin heavy constant gamma 2B | | | |  |  |  |  |
|  |  | | Ighg3 | Immunoglobulin heavy constant gamma 3 | | | |  |  |  |  |
|  |  | | Pld4 | phospholipase D family, member 4 | | |  |  |  |  |  |
|  |  | | Pros1 | protein S (alpha) | |  |  |  |  |  |  |
|  |  | | Hsp90aa1 | heat shock protein 90, alpha (cytosolic), class A member 1 | | | | |  |  |  |
|  |  | | Ighv2-6-8 | immunoglobulin heavy variable 2-6-8 | | |  |  |  |  |  |
|  |  | | Ighv2-9-1 | immunoglobulin heavy variable 2-9-1 | | |  |  |  |  |  |
|  |  | | Ighv7-1 | immunoglobulin heavy variable 7-1 | | |  |  |  |  |  |
|  |  | | Ighv2-6 | immunoglobulin heavy variable 2-6 | | |  |  |  |  |  |
|  |  | | Ighv2-9 | immunoglobulin heavy variable 2-9 | | |  |  |  |  |  |
|  |  | | Ighv2-5 | immunoglobulin heavy variable 2-5 | | |  |  |  |  |  |
|  |  | | Ighv2-7 | immunoglobulin heavy variable 2-7 | | |  |  |  |  |  |
|  |  | | Ighv2-2 | immunoglobulin heavy variable 2-2 | | |  |  |  |  |  |
|  |  | | Ighv2-3 | immunoglobulin heavy variable 2-3 | | |  |  |  |  |  |
|  |  | | Ighv7-2 | immunoglobulin heavy variable 7-2 | | |  |  |  |  |  |
|  |  | | Traf3 | TNF receptor-associated factor 3 | | |  |  |  |  |  |
|  |  | | Cd47 | CD47 antigen (Rh-related antigen, integrin-associated signal | | | | | |  |  |
|  |  | | Siva1 | SIVA1, apoptosis-inducing factor | | |  |  |  |  |  |
|  |  | | Arl6 | ADP-ribosylation factor-like 6 | | |  |  |  |  |  |
|  |  | | Alcam | activated leukocyte cell adhesion molecule | | | |  |  |  |  |
|  |  | | Boc | biregional cell adhesion molecule-related/down-regulated by | | | | | | | |
|  |  | | Stx19 | syntaxin 19 |  |  |  |  |  |  |  |
|  |  | | Jag2 | jagged 2 |  |  |  |  |  |  |  |
|  |  | | Olfr181 | olfactory receptor 181 | |  |  |  |  |  |  |
| Total Unique genes | **411** | |  |  |  |  |  |  |  |  |  |
